# Epigenetic regulation of cell fate reprogramming in aging and disease: A predictive computational model

**DOI:** 10.1371/journal.pcbi.1006052

**Published:** 2018-03-15

**Authors:** Núria Folguera-Blasco, Elisabet Cuyàs, Javier A. Menéndez, Tomás Alarcón

**Affiliations:** 1 Centre de Recerca Matemàtica, Edifici C, Campus de Bellaterra, Bellaterra (Barcelona), Spain; 2 Departament de Matemàtiques, Universitat Autònoma de Barcelona, Bellaterra (Barcelona), Spain; 3 Molecular Oncology Group, Girona Biomedical Research Institute (IDIBGI), Girona, Spain; 4 MetaboStem, Barcelona, Spain; 5 ProCURE (Program Against Cancer Therapeutic Resistance), Metabolism and Cancer Group, Catalan Institute of Oncology, Girona, Spain; 6 ICREA, Pg. Lluís Companys 23, Barcelona, Spain; 7 Barcelona Graduate School of Mathematics (BGSMath), Barcelona, Spain; Temple University, UNITED STATES

## Abstract

Understanding the control of epigenetic regulation is key to explain and modify the aging process. Because histone-modifying enzymes are sensitive to shifts in availability of cofactors (e.g. metabolites), cellular epigenetic states may be tied to changing conditions associated with cofactor variability. The aim of this study is to analyse the relationships between cofactor fluctuations, epigenetic landscapes, and cell state transitions. Using Approximate Bayesian Computation, we generate an ensemble of epigenetic regulation (ER) systems whose heterogeneity reflects variability in cofactor pools used by histone modifiers. The heterogeneity of epigenetic metabolites, which operates as regulator of the kinetic parameters promoting/preventing histone modifications, stochastically drives phenotypic variability. The ensemble of ER configurations reveals the occurrence of distinct epi-states within the ensemble. Whereas resilient states maintain large epigenetic barriers refractory to reprogramming cellular identity, plastic states lower these barriers, and increase the sensitivity to reprogramming. Moreover, fine-tuning of cofactor levels redirects plastic epigenetic states to re-enter epigenetic resilience, and vice versa. Our ensemble model agrees with a model of metabolism-responsive loss of epigenetic resilience as a cellular aging mechanism. Our findings support the notion that cellular aging, and its reversal, might result from stochastic translation of metabolic inputs into resilient/plastic cell states via ER systems.

## Author summary

Cell reprogramming, a process that allows differentiated cells to re-acquire stem-like properties, is increasingly considered a critical phenomenon in tissue regeneration, aging and cancer. In light of the importance of metabolism in controlling cell fate, we designed a computational model capable of predicting the likelihood of cell reprogramming in response to changes in aging-related metabolites. Our predictive mathematical model improves our understanding of how pathological processes that involve changes in cell plasticity, such as cancer, might be accelerated or attenuated by means of metabolic reprogramming.

## Introduction

Aging is associated with profound changes in the epigenome involving large disturbances of the epigenetic landscape and genome architecture [1, 2]. Studies in model organisms have not only revealed the complex changes occurring in chromatin structure and functioning during aging, but also the remarkable plasticity of age-associated epigenetic marks [3–5]. Thus, whereas epigenetic alterations in DNA methylation, post-translational modification (PTM) of histones and chromatin remodelling are considered highly conserved hallmarks of aging [4, 6], the ability of cellular reprogramming-driven epigenetic remodelling to ameliorate age-associated phenotypes has been described recently. This finding unequivocally supports the causative role of epigenetic dysregulation as a driver of aging [7]. The reversible nature of epigenetic regulation of aging is receiving increasing attention as it might offer a revolutionary strategy to simultaneously delay or reverse a spectrum of diseases, including cancer, clustered in older individuals [8, 9]. A mechanistic understanding of the dependence and inter-relationship between aging and the functional status of specific epigenetic modifiers, for example histone demethylases (HDMs) and histone deacetylases (HDACs), is largely lacking.

There is an increasing awareness of the relationship between epigenetic modifiers and metabolism. Common metabolites of intermediary metabolism, such as acetyl-CoA, NAD+, *α*-ketoglutarate, succinate, FAD, ATP or S-adenosylmethionine, drive epigenetic processes by directly regulating epigenetic modifiers. The usage of these intermediates as substrates and regulators of chromatin-modifying enzymes provides a direct link between the metabolic state of the cell and epigenetics [10–17]. However, it remains intriguing how aging-related changes in cellular metabolism (e.g., loss of NAD homeostasis [18–20]) might control the layers of epigenetic instructions that influence cell fate without involving changes in the DNA sequence.

The capacity of the chromatin structure to affect cellular identity and cellular state transitions can differ as a function of metabolic conditions that change during aging. However, the possibility that cellular aging might result from the stochastic translation of metabolic signals into cellular epigenetic states has not been formally evaluated.

In this paper, we explore the causative relationship between cofactor (e.g. metabolite) variability and chromatin modification state underpinning the aging-associated loss of epigenetic resilience, which leads to a gain of more plastic cell and tissue features. This fact might predispose aging tissues to cancer [21, 22]. To this end, we generated an ensemble of epigenetic regulation (ER) systems by means of Approximate Bayesian Computation (ABC) whose heterogeneity reflects the inhomogeneous abundance of cofactors used by epigenetic modifiers. By analysing the robustness of ER systems in response to the regulation of HDM and HDAC activity, we present a model of ER capable of formulating strategies aimed at modifying the aging process and the aging-dependency of cancer, based on the control of epigenetic resilience and plasticity.

Recent advances in experimental determination of the mechanisms of ER have triggered an interest in developing mathematical models capable of reducing their intrinsic complexity to essential components such as ER of gene expression [17, 23–27] and epigenetic memory [24, 25, 27–32]. For comprehensive reviews, we refer the readers to [25, 27]. In order to put our model into context, we briefly summarise the current state of the art in ER modelling.

Models of ER were originally formulated in order to shed light onto the mechanisms of epigenetic memory; since DNA during cell cycle is duplicated and, therefore, the epigenetic marks diluted, early ER models were aimed at explaining how epigenetic-regulatory states remain stable upon cell division and transmitted to daughter cells. Such models must satisfy two essential properties, namely, they must be bistable, i.e., each steady state corresponding to an alternative epigenetic state, and the basin of attraction of such states must allow that large perturbations of the ER systems undergoing DNA replication should not change the epigenetic state thus allowing mitotic heritability [29].

Dodd et al. [28] developed the first of such ER models. The authors considered a region of DNA consisting of N nucleosomes, each assumed to be in either of three states, namely unmodified (*U*), methylated (*M*), and acetylated (*A*). Because modifying and de-modifying enzymes carry out nucleosome modifications and removal of marks, a crucial ingredient of the model by Dodd et al. [28] is that histone-modifying enzymes are recruited by modified nucleosomes, thereby providing the necessary positive feed-back for the system to be bistable. However, recruitment based on next-neighbours interactions is not enough to produce robust bistability. Long-range correlations are necessary.

The model by Dodd et al. [28] has been modified and extended in several ways [31]. Sneppen and Dodd have successfully applied the same ideas [32] to modelling the patterns of epigenetic regulation in CpG islands [33]. Another interesting feature of the model developed by Sneppen and Dodd [31] is that medium-length correlations are provided by the size of nucleosomes, which allows relaxing the requirement for recruited demethylation. Angel et al. [30] have proposed an ER model to explain quantitative epigenetic control associated with the phenomenon of vernalisation, i.e. the perception and epigenetic memory of a period of cold temperatures to initiate flowering later. This model is capable of reproducing both the patterns of flowering locus C (*FLC*) and the quantitative dependence with respect to the duration of the exposition to low temperatures.

Besides the issue of maintaining stable epigenetic memory, recent efforts have been dedicated to the study of the regulation of epigenetic modifications by transcription factors [23, 26]. Based on the experimental observation that transcription factors (TFs) can recruit histone-modifying enzymes, Sneppen et al. [23] proposed a model where transcription factors are coupled to ER. A similar approach, although with rather significant differences, has been recently proposed by Berry et al. [26]. An essential feature of this model is the proposed feedback between transcription and epigenetic chromatin modification: activation of transcription depends on the balance between positive and negative modifications, and, in turn, each passage of RNA polymerase II, which is modelled as a discrete event, causes demethylation (see [26] for details). An important feature that distinguishes this model from its predecessors is the assumption of next-neighbour recruitment as exclusively opposed to long-distance recruitment.

Bintu et al. [24] have recently proposed a more phenomenological ER model capable of explaining experimental data obtained by using a reporter gene that expresses a fluorescent protein with induced recruitment of a number of epigenetic-modifying enzymes. The model by Bintu et al. [24] considers active, reversible silent, and irreversible silent states and is able to predict the rates of transition between states.

## Materials and methods

In this Section, we provide an account of our stochastic model of epigenetic regulation of gene expression which extends our previous work [17]. Our model belongs to a family of models which consider that single unmodified (*U*) loci can be modified so as to acquire positive (*A*) or negative (*M*) marks. A positive feedback mechanism is introduced whereby *M* marks help to both add more *M* marks and remove *A* marks from neighbouring loci. The positive marks are assumed to be under the effects of a similar positive reinforcement mechanism [27, 28].

### Stochastic model of epigenetic regulation

The stochastic model of epigenetic regulation is formulated in terms of the associated Chemical Master Equation (CME), which, in general, is given by:

$$\frac{\partial P(X,t)}{\partial t}=\sum_{i}\left(W_{i}\left(X-r_{i}\right)P\left(X-r_{i},t\right)-W_{i}\left(X\right)P\left(X,t\right)\right)$$ (1)

where **X** = (*X*_1_, …, *X*_*n*_) is the vector containing the number of molecules of each molecular species at time t, *W*_*i*_(**X**) is the transition rate corresponding to reaction channel *i* and **r**_*i*_ is a vector whose entries denote the change in the number of molecules of each molecular species when reaction channel *i* fires up, i.e. *P*(**X**(*t* + Δ*t*) = **X**(*t*) + **r**_*i*_|**X**(*t*)) = *W*_*i*_(**X**)Δ*t*. Our model (see Table 1) is based on the stochastic models by Dodd et al. [28] and Menéndez et al. [34].

**Table 1:** Random processes and their transition rates. Reaction numbers correspond to the enumeration in Section Stochastic model of epigenetic regulation. *X*_1_, *X*_2_, *X*_3_, *X*_4_, *X*_5_, *X*_6_, and *X*_7_ are the numbers of unmodified nucleosomes, methylated nucleosomes, acetylated nucleosomes, HDM molecules, methylated nucleosome-HDM complexes, HDAC enzyme molecules, and acetylated nucleosome-HDAC enzyme complexes, respectively.

Transition rate	*r*	Event
*W*_1_(*x*) = *k*_1_*X*_2_*X*_4_	*r*_1_ = (0, −1, 0, −1, +1, 0, 0)	Formation of M-nucleosome-HDM enzyme complex (unrecruited); Reaction 1
*W*_2_(*x*) = *k*_2_*X*_5_	*r*_2_ = (0, +1, 0, +1, −1, 0, 0)	M-nucleosome-HDM enzyme complex splits (unrecruited); Reaction 1
*W*_3_(*x*) = *k*_3_*X*_5_	*r*_3_ = (+1, 0, 0, +1, −1, 0, 0)	Demethylation and HDM enzyme release (unrecruited); Reaction 1
*W*_4_(*x*) = *k*_4_*X*_2_*X*_3_*X*_4_	*r*_4_ = (0, −1, 0, −1, +1, 0, 0)	Formation of M-nucleosome-HDM enzyme complex (recruited); Reaction 1
*W*_5_(*x*) = *k*_5_*X*_3_*X*_5_	*r*_5_ = (0, +1, 0, +1, −1, 0, 0)	M-nucleosome-HDM enzyme complex splits (recruited); Reaction 1
*W*_6_(*x*) = *k*_6_*X*_3_*X*_5_	*r*_6_ = (+1, 0, 0, +1, −1, 0, 0)	Demethylation and HDM enzyme release (recruited); Reaction 1
*W*_7_(*x*) = *k*_7_*X*_1_	*r*_7_ = (−1, +1, 0, 0, 0, 0, 0)	Methylation (unrecruited); Reaction 2
*W*_8_(*x*) = *k*_8_*X*_1_*X*_2_	*r*_8_ = (−1, +1, 0, 0, 0, 0, 0)	Methylation (recruited); Reaction 2
*W*_9_(*x*) = *k*_9_*X*_3_*X*_6_	*r*_9_ = (0, 0, −1, 0, 0, −1, +1)	Formation of A-nucleosome-HDAC enzyme complex (unrecruited); Reaction 3
*W*_10_(*x*) = *k*_10_*X*_7_	*r*_10_ = (0, 0, +1, 0, 0, +1, −1)	A-nucleosome-HDAC enzyme complex splits (unrecruited); Reaction 3
*W*_11_(*x*) = *k*_11_*X*_7_	*r*_11_ = (+1, 0, 0, 0, 0, +1, −1)	Deacetylation and HDAC enzyme release (unrecruited); Reaction 3
*W*_12_(*x*) = *k*_12_*X*_3_*X*_2_*X*_6_	*r*_12_ = (0, 0, −1, 0, 0, −1, +1)	Formation of A-nucleosome-HDAC enzyme complex (recruited); Reaction 3
*W*_13_(*x*) = *k*_13_*X*_7_*X*_2_	*r*_13_ = (0, 0, +1, 0, 0, +1, −1)	A-nucleosome-HDAC enzyme complex splits (recruited); Reaction 3
*W*_14_(*x*) = *k*_14_*X*_7_*X*_2_	*r*_14_ = (+1, 0, 0, 0, 0, +1, −1)	Deacetylation and HDAC enzyme release (recruited); Reaction 3
*W*_15_(*x*) = *k*_15_*X*_1_	*r*_15_ = (−1, 0, +1, 0, 0, 0, 0)	Acetylation (unrecruited); Reaction 4
*W*_16_(*x*) = *k*_16_*X*_1_*X*_3_	*r*_16_ = (−1, 0, +1, 0, 0, 0, 0)	Acetylation (recruited); Reaction 4

Dodd et al. [28] consider that direct transitions between *M* and *A* are very unlikely. Instead, they assume that transitions occur in a linear sequence given by *M* ⇌ *U* ⇌ *A*. They further put forward the hypothesis that such nucleosome modifications are of two types, namely, recruited and unrecruited. Mathematically, recruited modifications are represented by non-linear dependence on the number of *M*-nucleosomes and *A*-nucleosomes of the corresponding transition rates (see Table 1).

Specifically, the reactions involved in our model are:

1. HDM-mediated demethylation: *M* + *HDM* ⇆ *C*_*M*_ → *U* + *HDM*
2. Methylation: *U* → *M*
3. HDAC-mediated deacetylation: *A* + *HDAC* ⇆ *C*_*A*_ → *U* + *HDAC*
4. Acetylation: *U* → *A*

All these reactions can be both recruited or unrecruited. The associated reactions rates are reported in Table 1.

We consider the scenario where both hyper-(hypo-)abundance of *A* (*M*) marks allows for genes to be expressed, insofar the associated transcription factors are present [10]. On the contrary, we associate hypo-(hyper-)abundance of *A* (*M*) marks with silent states where genes are not expressed even in the presence of the appropriate transcription factors. We here focus on the conditions for bistability to arise and the robustness of the associated *open* and *closed* states particularly in connection with the abundance or activity of HDMs and HDACs. Our aim is to analyse the effects of varying the concentration of these enzymes as well as possible synergies between them.

In more detail, we focus our analysis on plastic behaviour of the epigenetic regulatory states when the activity of histone-modifying enzymes (HMEs) is down-regulated against the background of heterogeneity due to variability in the pool of cofactors for chromatin-modifying enzymes. We proceed by first defining a base-line scenario (which we categorise as *normal cell*) in which the associated epigenetic regulatory system is such that, for average values of HDM and HDAC activities, the differentiation-promoting gene ER is open and the pluripotency-promoting gene ER is closed. We then proceed to generate an ensemble of ER systems that satisfy the requirements imposed by this base-line scenario; the necessary variability to generate this ensemble is provided by heterogeneity in abundance of epigenetic cofactors. Analysis of this ensemble reveals that the requirements of the base line scenario restrict the values of a few parameters only, leaving ample flexibility to fix the rest of them. This behaviour is typical of the so-called *sloppy models* [35], where available data constrains a limited number of parameters (or parameter combinations), the system being robust to the choice of a large number of model parameters. In our case, this feature is absolutely essential since, nested within this heterogeneous ensemble of ER systems, there exists a sub-ensemble of plastic ER systems.

### Mean-field limit and quasi-steady state approximation

In order to gain some insight into the behaviour of the stochastic ER model, we analyse its mean-field limit regarding time scale separation and the quasi-steady state approximation. For a full account of the technicalities we refer the reader to our previous work [36, 37].

The mean-field equations, which describe the time evolution of the ensemble average of the variables *X*_*i*_, associated to the stochastic system with rates given in Table 1 are:

$$\frac{dQ_{i}}{dt}=\sum_{j=1}^{16}r_{j,i}W_{j}\left(Q\right)$$ (2)

where **Q** is a vector whose entries, *Q*_*i*_, are *Q*_*i*_ ≡ 〈*X*_*i*_〉. In order to proceed further, we assume that the variables describing the system are divided into two groups according to their characteristic scales. More specifically, we consider the situation where the subset of chemical species *X*_*i*_, with *i* = 1, 2, 3, scale as *X*_*i*_ = *Sx*_*i*_, where *x*_*i*_ = *O*(1), whilst the remaining species are such that *X*_*i*_, with *i* = 4, 5, 6, 7, scale as *X*_*i*_ = *Ex*_*i*_, where *x*_*i*_ = *O*(1). Key to our approach is the further assumption that *S* and *E* must be such that $\epsilon =\frac{E}{S}⪡1$. The averaged variables, *Q*_*i*_, are similarly divided into two groups: slow variables, i.e. *Q*_*i*_ = *Sq*_*i*_ (*i* = 1, 2, 3), and fast variables, i.e. *Q*_*i*_ = *Eq*_*i*_ (*i* = 4, 5, 6, 7).

Under this rescaling, we define the following scale transformation for the transition rates in Table 1: *W*_*j*_(**Q**) = *k*_4_*S*^2^*Eω*_*j*_(**q**). We further rescale the time variable so that a dimensionless variable, *τ*, is defined as *τ* = *k*_4_*SEt*. It is now straightforward to verify that, upon rescaling, the mean-field equations become:

$$\frac{dq_{i}}{d\tau }=\sum_{j=1}^{16}r_{j,i}\omega_{j}\left(q\right),\;i=1,2,3,$$ (3)

$$\epsilon \frac{dq_{i}}{d\tau }=\sum_{j=1}^{16}r_{j,i}\omega_{j}\left(q\right),\;i=4,5,6,7.$$ (4)

with *ϵ* = *E*/*S*.

If *ϵ* = *E*/*S* ≪ 1 holds, Eqs (3) and (4) naturally display multiple scales structure, which we will exploit to simplify our analysis by means of a quasi-steady state approximation (QSSA) [38], which is given by:

$$\begin{array}{ccc} & & \frac{dq_{1}}{d\tau }=e_{HDM}\frac{\left(\kappa_{1}+q_{3}\right)\left(\kappa_{3}+\kappa_{6}q_{3}\right)q_{2}}{\left(\kappa_{2}+\kappa_{3}\right)+\left(\kappa_{1}+q_{3}\right)q_{2}+\left(\kappa_{5}+\kappa_{6}\right)q_{3}}+e_{HDAC}\frac{\left(\kappa_{9}+\kappa_{12}q_{2}\right)\left(\kappa_{11}+\kappa_{14}q_{2}\right)q_{3}}{\left(\kappa_{10}+\kappa_{11}\right)+\left(\kappa_{9}+\kappa_{12}q_{2}\right)q_{3}+\left(\kappa_{13}+\kappa_{14}\right)q_{2}}\\ & & \;-\left(\kappa_{8}q_{2}+\kappa_{7}+\kappa_{16}q_{3}+\kappa_{15}\right)q_{1} \end{array}$$ (5)

$$\frac{dq_{2}}{d\tau }=-e_{HDM}\frac{\left(\kappa_{1}+q_{3}\right)\left(\kappa_{3}+\kappa_{6}q_{3}\right)q_{2}}{\left(\kappa_{2}+\kappa_{3}\right)+\left(\kappa_{1}+q_{3}\right)q_{2}+\left(\kappa_{5}+\kappa_{6}\right)q_{3}}+\left(\kappa_{8}q_{2}+\kappa_{7}\right)q_{1}$$ (6)

$$\frac{dq_{3}}{d\tau }=-e_{HDAC}\frac{\left(\kappa_{9}+\kappa_{12}q_{2}\right)\left(\kappa_{11}+\kappa_{14}q_{2}\right)q_{3}}{\left(\kappa_{10}+\kappa_{11}\right)+\left(\kappa_{9}+\kappa_{12}q_{2}\right)q_{3}+\left(\kappa_{13}+\kappa_{14}\right)q_{2}}+\left(\kappa_{16}q_{3}+\kappa_{15}\right)q_{1}$$ (7)

$$q_{4}=e_{HDM}\frac{\kappa_{2}+\kappa_{3}+\left(\kappa_{5}+\kappa_{6}\right)q_{3}}{\left(\kappa_{2}+\kappa_{3}\right)+\left(\kappa_{1}+q_{3}\right)q_{2}+\left(\kappa_{5}+\kappa_{6}\right)q_{3}}$$ (8)

$$q_{5}=e_{HDM}\frac{\left(\kappa_{1}+q_{3}\right)q_{2}}{\left(\kappa_{2}+\kappa_{3}\right)+\left(\kappa_{1}+q_{3}\right)q_{2}+\left(\kappa_{5}+\kappa_{6}\right)q_{3}}$$ (9)

$$q_{6}=e_{HDAC}\frac{\kappa_{10}+\kappa_{11}+\left(\kappa_{13}+\kappa_{14}\right)q_{2}}{\left(\kappa_{10}+\kappa_{11}\right)+\left(\kappa_{9}+\kappa_{12}q_{2}\right)q_{3}+\left(\kappa_{13}+\kappa_{14}\right)q_{2}}$$ (10)

$$q_{7}=e_{HDAC}\frac{\left(\kappa_{9}+\kappa_{12}q_{2}\right)q_{3}}{\left(\kappa_{10}+\kappa_{11}\right)+\left(\kappa_{9}+\kappa_{12}q_{2}\right)q_{3}+\left(\kappa_{13}+\kappa_{14}\right)q_{2}}$$ (11)

where the re-scaled parameters *κ*_*j*_ are defined in Table 2, and the conservation laws *q*_4_(*τ*) + *q*_5_(*τ*) = *e*_*HDM*_ and *q*_6_(*τ*) + *q*_7_(*τ*) = *e*_*HDAC*_ hold. These conservation laws account for the fact that the total number of enzyme molecules, i.e. the enzyme molecules in their free form and those forming a complex must be constant. Hence, the quantities *e*_*HDM*_ and *e*_*HDAC*_ are defined as $e_{HDM}=\frac{z_{0}}{E}$ and $e_{HDAC}=\frac{v_{0}}{E}$, respectively, where *z*_0_ and *v*_0_ are the numbers of HDM and HDAC enzyme molecules, respectively. *E* is the characteristic scale (i.e. average) of abundance of the histone-modifying enzymes which, for simplicity, has been taken to have the same value for both HDMs and HDACs. This result opens interesting avenues to investigate, since both oncometabolic transformation and aging appear to reduce the number of both types of enzymes. Our theory thus allows us in a natural manner to explore the effects of these anomalies on the stability of epigenetic regulatory states.

**Table 2:** Mean-field limit dimensionless parameters.

Dimensionless parameters
*ϵ* = *E*/*S*, *κ*_1_ = *k*_1_/(*k*_4_*S*), *κ*_2_ = *k*_2_/(*k*_4_*S*^2^), *κ*_3_ = *k*_3_/(*k*_4_*S*^2^)
*κ*_5_ = *k*_5_/(*k*_4_*S*), *κ*_6_ = *k*_6_/(*k*_4_*S*), *κ*_7_ = *k*_7_/(*k*_4_*SE*), *κ*_8_ = *k*_8_/(*k*_4_*E*)
*κ*_9_ = *k*_9_/(*k*_4_*S*), *κ*_10_ = *k*_10_/(*k*_4_*S*^2^), *κ*_11_ = *k*_11_/(*k*_4_*S*^2^), *κ*_12_ = *k*_12_/(*k*_4_)
*κ*_13_ = *k*_13_/(*k*_4_*S*), *κ*_14_ = *k*_14_/(*k*_4_*S*), *κ*_15_ = *k*_15_/(*k*_4_*SE*), *κ*_16_ = *k*_16_/(*k*_4_*E*)

### Parameter values and ensemble generation

#### Viability conditions and reference parameter values

**Fig 1:**
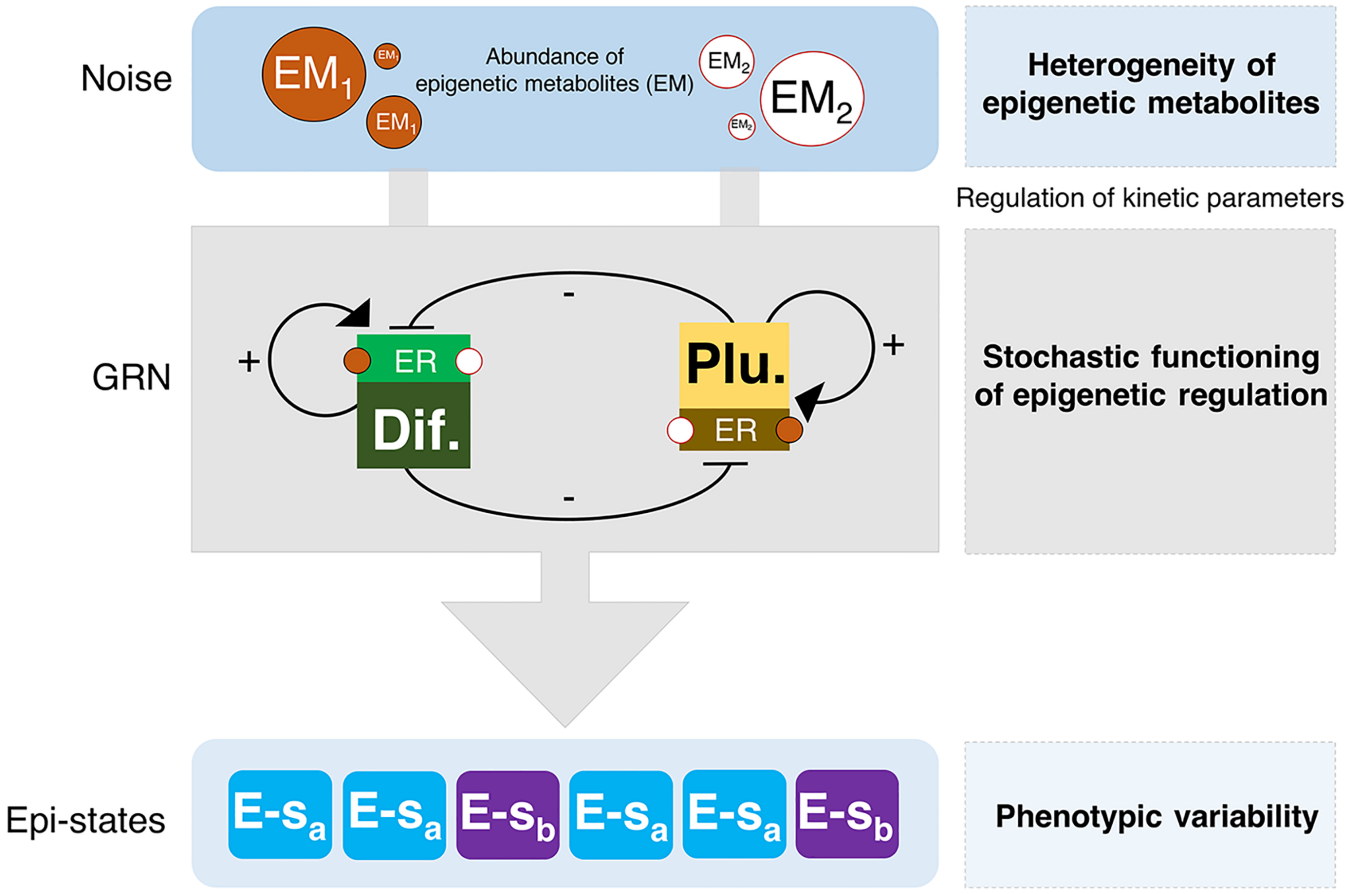
A stochastic model of aging metabolism-regulated cell fate. Schematic representation of the minimal gene regulatory network (GRN) considered in our stochastic model of epigenetic regulation (ER), consisting of a coupled pluripotency and differentiation modules. The heterogeneity of epigenetic metabolites (EM), which operates as regulator of the kinetic parameters promoting/preventing the functioning of histone modifiers, stochastically drives phenotypic variability (epi-states). Arrows denote activation and blunt-ended lines denote inhibitory interactions.

In order to define our viability conditions unambigously, we restrict our discussion to the context of the gene regulatory network used in our previous study [17], i.e. a network of mutually repressive differentiation genes and mutually reinforcing pluripotency genes, as shown in Fig 1, with further mutual inhibition between differentiation and pluripotency genes [39]. Within such context, the phenotype of a normal somatic, differentiated cell demands that those genes promoting pluripotent behaviour and/or proliferation should be silent, whereas genes promoting differentiation and quiescent behaviour should be active. We therefore consider that our epigenetic regulatory (ER) systems are composed of two replicas of the stochastic epigenetic regulation model, Section Stochastic model of epigenetic regulation, with two sets of parameter values, associated with differentiation-promoting and pluripotency-promoting genes. For the remainder of this manuscript, an *open* epigenetic state will refer to a steady state of the system where *q*_1_ ≃ *q*_2_ ≃ 0 and *q*_3_ ≃ 1 (highly acetylated). A *closed* or *silent* epigenetic state is associated with *q*_1_ ≃ 0, *q*_2_ ≃ 1 and *q*_3_ ≃ 0 at equilibrium (highly methylated). The biological rational for these definitions, based on recent experimental evidence, is as follows. PTM of individual histones, such as acetylation and methylation, plays pivotal roles in the epigenetic regulation of gene expression through chromatin structure changes. Histone acetylation is generally associated with a chromatin structure that is open and therefore accessible to transcription factors and, therefore, gene activation [2, 40, 41]. Histone methylation is linked to either active or repressed genes, depending on the residue that is being modified (e.g., H3K4me3 mark is associated with active promoters whereas H3K27me3 and H3K9me2/3 are associated with repressed regulatory regions). Although it is likely that the sum of numerous PTMs within regulatory regions determine the transcriptional state of a specific set of genes, for practical reasons epigenetic studies usually involve profiling of one or a couple of well-established histone modifications. Nevertheless, the silent/closed chromatin state is associated with low levels of acetylation and high levels of certain methylated sites. Our computational model acknowledges not only that, during aging, the abundance and activity of enzymes in charge of adding and removing histone changes, but also the complexity arising from the fact that chromatin-modifying enzymes for both activating and repressive histone marks require metabolites.

For each component of the ER system (differentiation and pluripotency epigenetic regulation), we have set the parameters *κ*_*j*_ so that they satisfy the following general viability conditions, namely, (i) when *e*_*HDM*_ = *e*_*HDAC*_ = 1, the regulatory system is mono-stable (stable open chromatin state for the differentiation ER, stable closed chromatin state for the pluripotency ER), and (ii) for *e*_*HDM*_ < 1, *e*_*HDAC*_ < 1 both the differentiation and pluripotency ER exhibit a bistable regime. Reference (default) parameter values satisfying the viability conditions for the different scenarios described later on are given in the Section II in S1 File.

#### Ensemble generation

**Fig 2:**
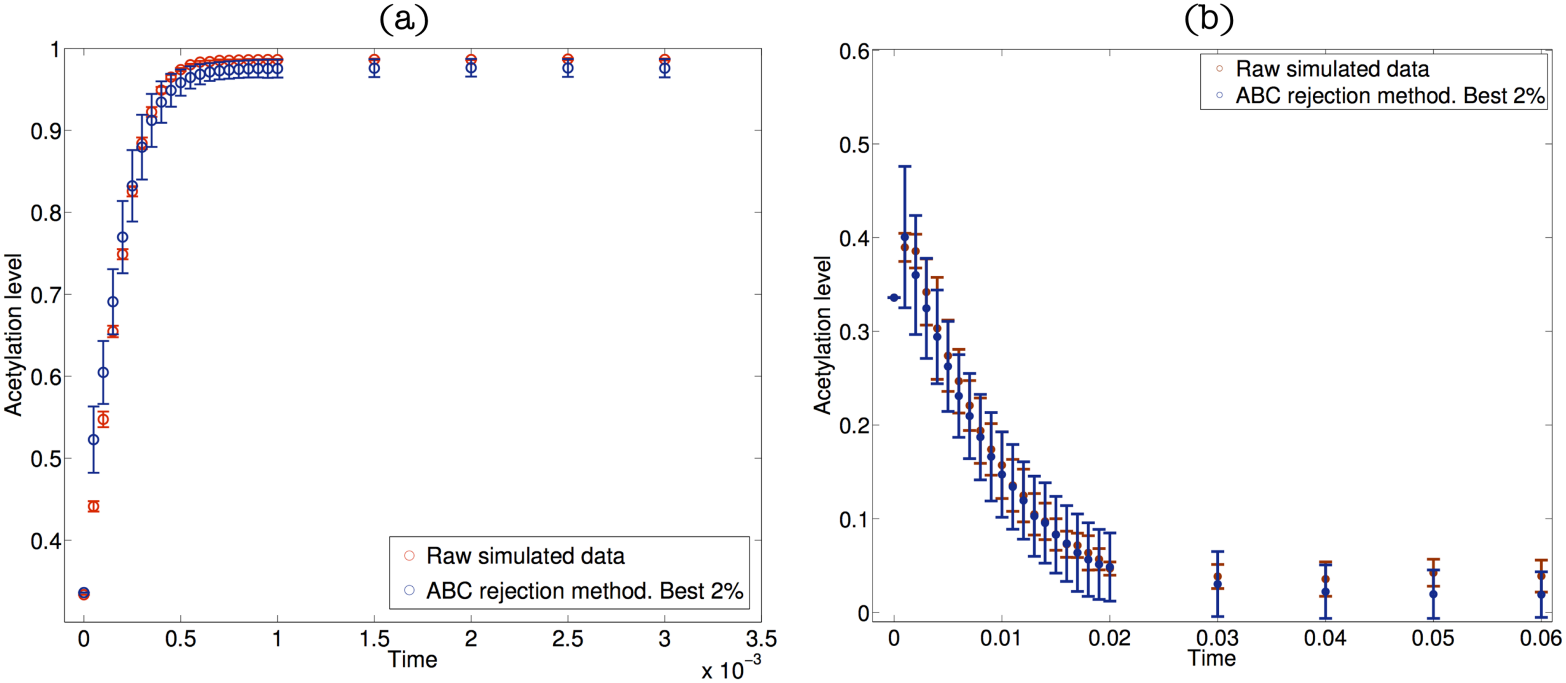
Plot (a) shows results regarding the parametric sensitivity analysis of the epigenetic regulatory system for the differentiation-regulating gene. Plot (a) shows the comparison between the raw simulated data and the ABC ensemble average, limited to the 200 ABC parameter sets that best fit the data. Plot (b), idem for the pluripotency-regulating gene. Raw simulated data is generated by using the SSA on the model defined by the rates shown in Table 1 with parameter values given in Tables A and B in S1 File.

Beyond the behaviour of the ER system for the reference parameter sets (Tables A & B and Tables C & D in S1 File), we have generated an ensemble of ER systems to analyse the robustness of the different scenarios we analyse later on in Section Results. Such ensemble is generated using approximate Bayesian computation (ABC). Details are provided in full in Section Heterogeneity and robustness of the refractory and plastic scenarios and in Section III in S1 File. The generated kinetic rate constants are dimensionless, i.e. they are relative to a global scale associated to *k*_4_ (see Table 2). Such feature implies that there is an undetermined time scale in our system associated with the (inverse of the) rate constant *k*_4_. This additional degree of freedom can be used to fit our model of epigenetic (de-)activation to particular data. Furthermore, the global time scale corresponding to the differentiation ER regulation (i.e. de-silencing dynamics, Fig 2(a)) need not coincide with the global time scale associated with the pluripotency ER system (i.e. silencing dynamics, Fig 2(b)). Therefore, our model has the capability of reproducing different systems characterised by different time scales as previously shown by Bintu et al. [24].

## Results

We now proceed to explore the behaviour of our system as the number of HDMs and HDACs vary relative to their average abundance against the background of variability provided by our ABC-ensemble approach.

### Variation in the abundance of HDM and HDAC drives epigenetic switch

**Fig 3:**
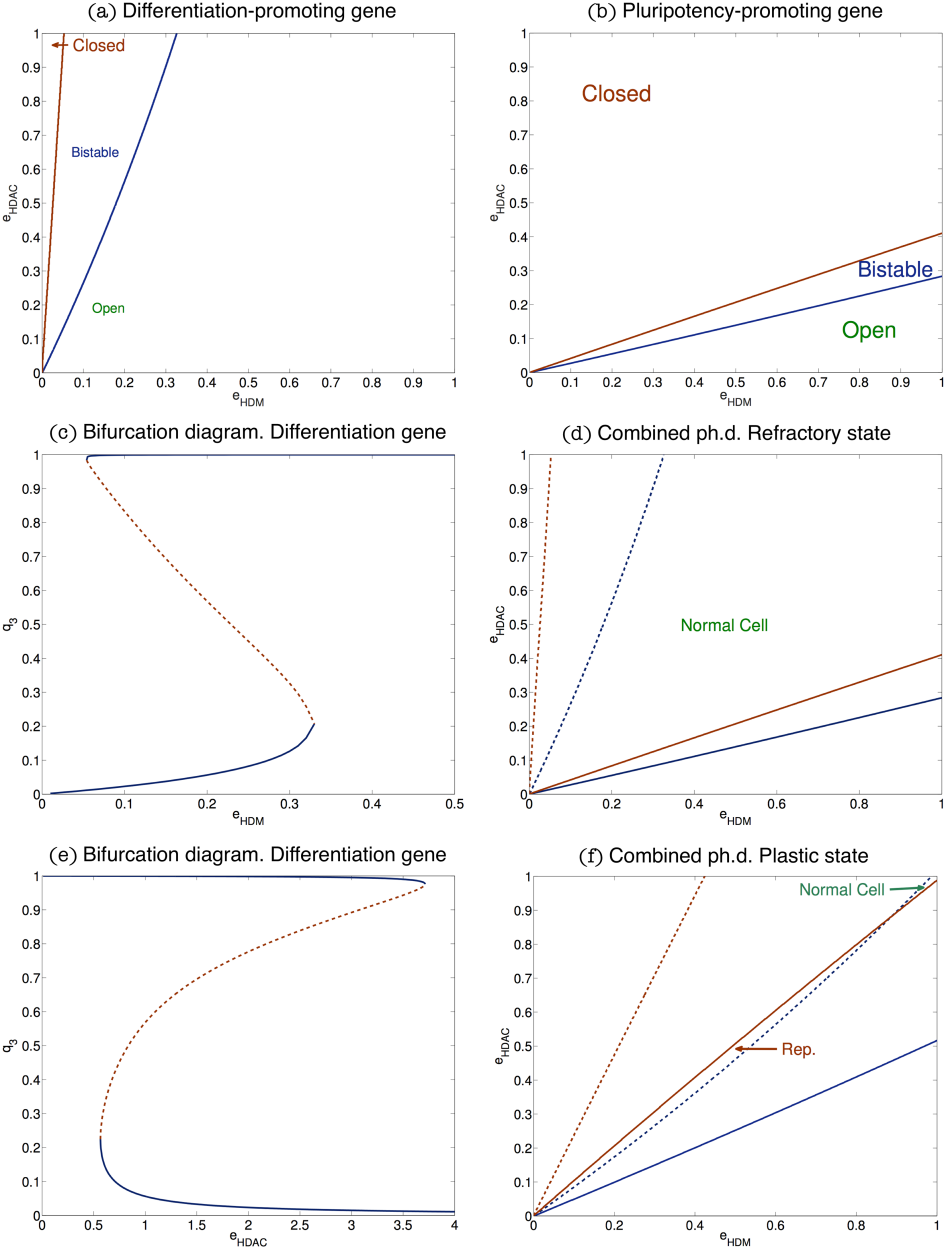
Plots (a) and (b) show the phase diagrams associated with the QSS approximation for the differentiation and pluripotency promoting genes, respectively. We examine the stability properties of the QSSA as when *e*_*HDM*_ and *e*_*HDAC*_ are varied. The system exhibits bistability in the region between the red and blue lines. In the region above the red line the only stable steady state is the closed state. By contrast, in the region below the blue line only the open steady state is stable. Parameters values are given in Table A in S1 File for the differentiation-promoting gene and Table B in S1 File for the pluripotency-promoting gene. Plots (d) and (f) show the combined phase diagram for both the differentiation-promoting and the pluripotency-promoting models of epigenetic regulation for two clinically relevant cases. In both plots, solid (dashed) lines correspond to the stability limits of the pluripotency(differentiation)-promoting gene. In plot (d), the region between the solid red line and the dashed blue line is associated with *normal cell* behaviour, i.e. open differentiation-promoting gene and silenced pluripotency-promoting gene, whereas in Plot (f), the region marked as *Rep*. is associated with epigenetic regulation configurations which facilitate cell reprogramming. Plot (d) shows a *refractory* epigenetic scenario and Plot (f) depicts a *plastic* scenario. Parameter values for Plot (d) as per Table A in S1 File (dashed lines) and Table B in S1 File (solid lines). Parameter values for Plot (f) are given in Table C in S1 File, and Table D in S1 File. Plots (c) and (e) show two bifurcation diagrams, i.e. two sections of Plot (a), corresponding to the differentiation-promoting gene, of the QSS approximation. Plot (c) corresponds to fixing *e*_*HDAC*_ = 1 and letting HDM activity to vary. Plot (e) examines the bifurcation properties of the system for *e*_*HDM*_ = 0.2 as HDAC concentration changes.

**Fig 4:**
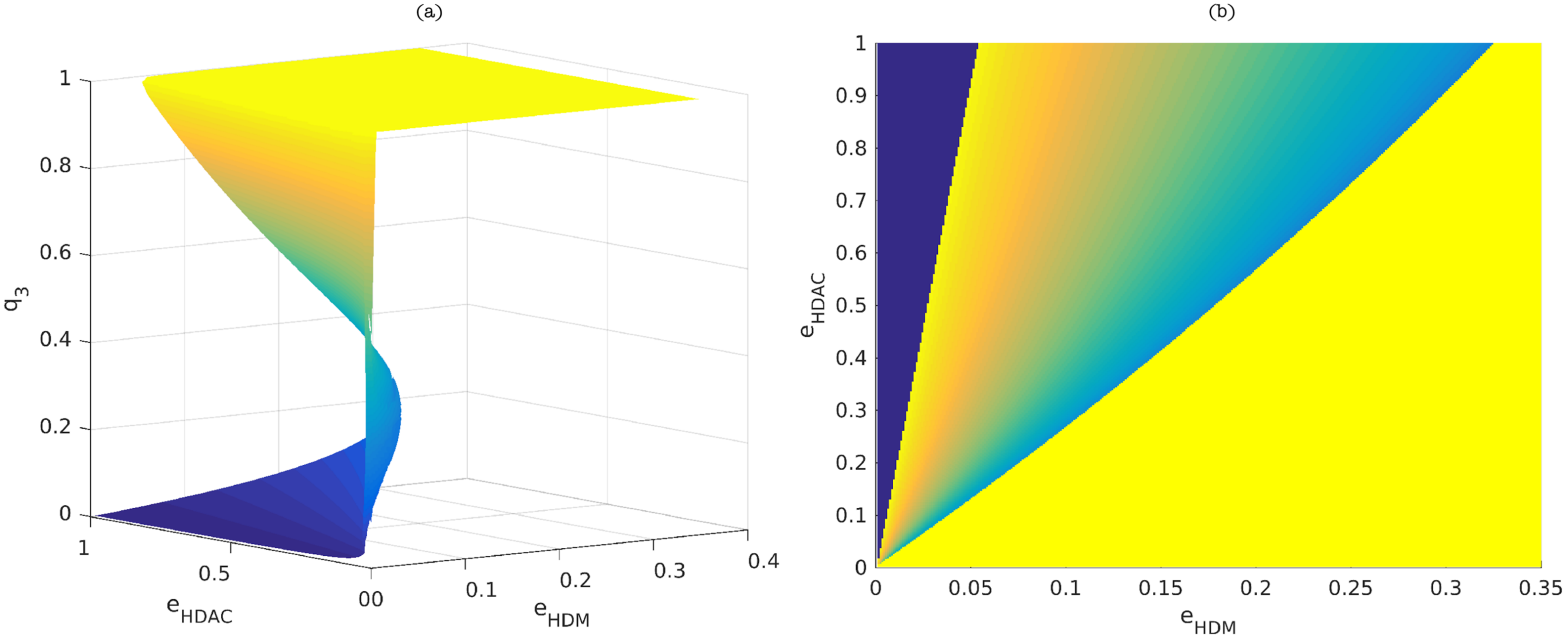
Plot (a) shows a 3D plot, where the *x*-axis represents *e*_*HDM*_, *y*-axis represents *e*_*HDAC*_ and the *z*-axis represents the steady state value of positive marks, *q*_3_. Depending on the *q*_3_ value, the system can be open (high value of *q*_3_), closed (low value of *q*_3_) or bistable (region where the two states coexist, together with an unstable state). Plot (b) represents a projection of the plot shown in (a) on the *xy*-plane. In this plot, we can again identify the three regions: closed (left region), bistable (middle region) and open (right region). These regions can be easily understood by matching the color of each region to the ones shown in Plot (a), which, in turn, can be related to levels of *q*_3_.

We first focus on a bifurcation analysis of the mean-field QSSA Eqs (5)–(11), to investigate the qualitative behaviour of the ER system as the relative abundances of HDMs and HDACs are varied. Results are shown in Fig 3(a) and 3(b). In particular, the phase space of both ER systems obtained by varying the parameters *e*_*HDM*_ and *e*_*HDAC*_. Both these diagrams display three differentiated regions: one in which the only stable steady-state is the one associated with a silenced gene, another one in which the only stable steady-state is the corresponding to an open gene, and a third one where the system is bistable. Fig 3(a) is associated with the differentiation-promoting gene, and Fig 3(b) corresponds to the pluripotency-promoting gene (parameters as per Table A, Table B in S1 File, respectively). In order to clarify the three regions (open, closed and bistable) displayed in Fig 3(a), a 3D plot is shown in Fig 4(a), where the vertical axis shows the level of positive marks (*q*_3_). This plot shows that the system dysplays bistable behaviour: depending on the parameter values *e*_*HDM*_ and *e*_*HDAC*_, the system may be both in the open state (high levels of *q*_3_, top of the plot), or in the closed state. Fig 4(b) displays the projection on the *xy*-plane of the plot shown in Fig 4(a), where we can clearly identify the three regions described in Fig 3(a).

A more detailed picture of the situation illustrated in Figs 3(a) and 4 is given in Fig 3(c), which shows the bifurcation diagram where *e*_*HDM*_, i.e. HDM concentration, is taken as the control parameter, whilst keeping *e*_*HDAC*_ constant. In particular we show the steady state value of *q*_3_, i.e. the variable with positive marks, as a function of HDM concentration. This allows to distinguish the three regions displayed in Fig 3(a). We observe, that a decrease in HDM makes the corresponding gene inaccessible to the transcription machinery (corresponding to the closed region, Fig 3(a)). As HDM concentration recovers, the system enters a bistable regime where both the active and silent states coexist (region marked as bistable in Fig 3(a)). Further increase of the demethylase concentration drives the system through a saddle-node bifurcation, beyond which the only stable steady-state is the active state (region labelled as open in Fig 3(a)). It is noteworthy that these results are in agreement with the oncometabolic transformation scenario associated with IDH mutations proposed by Thompson and co-workers [10, 42] in which downregulation of HDM activity locks differentiation genes into a silenced state which favours reprogramming of the differentiated state of somatic cells into a pluripotent phenotype [17]. The association between IDH mutations and cancer progression has been well established in the case of glioblastomas and acute myelogenous leukaemia [43–46].

In Fig 3(e), we show the bifurcation diagram associated with fixing *e*_*HDM*_ and varying *e*_*HDAC*_. Within the scenario we are considering, i.e. the epigenetic regulation of a differentiation-regulating gene, reduced HDAC concentration recovers the base-line state where the epigenetic regulatory machinery is set to the open state. As HDAC concentration recovers, the system enters a bistable regime in which both the active and silent states coexist. Further increase in HDAC activity locks the system into the close chromatin state so that the gene is silenced. This implies that reduced HDAC activity may help to rescue differentiation-regulating genes from the effects of IDH mutation.

Numerical results which verify the predictions of the bifurcation analysis are presented and discussed in Section I in S1 File.

### Mean-field analysis of the stochastic epigenetic regulation model: Refractory vs plastic scenario

We now proceed to analyse in more detail the implications of the bifurcation analysis, regarding robustness of the epigenetic regulatory state. In Fig 3(d), which shows the phase diagram of both modes of epigenetic regulation (differentiation- and pluripotency-promoting) in the same phase space, the region between the solid red line and the dashed blue line represents the part of the phase space where the differentiation genes are open and the pluripotency genes are closed (region marked as Normal Cell in Fig 3(d)). This sub-space is therefore associated with normal, differentiated somatic cells. As we have previously shown [17], efficient reprogramming requires both closed differentiation genes and open pluripotency genes. Such situation is not viable under the scenario shown in Fig 3(d) because these two conditions cannot hold simultaneously, which we therefore dubb as the *refractory scenario*.

By contrast, Fig 3(f) corresponds to a *plastic scenario*, where, under appropriate conditions, cells become poised for reprogramming. The main difference with the refractory scenario is the intersection between the bistability regions of both the differentiation regulator and the pluripotency gene. In Fig 3(f), the regime where both bistability regions overlap is the one between the red solid line and the blue dashed line (region marked as Rep. in Fig 3(f)). Within this region, since both genes are in the bistable epigenetic regulatory regime, it is possible to find the differentiation gene in its closed state and the pluripotency gene in the open state. Such situation makes reprogramming much more likely to occur [17] and therefore we identify this feature of the phase space with plastic behaviour. By driving the ER system into this region by means of down-regulation of both HDM and HDAC activity, cells become epigenetically poised to undergo reprogramming. This is consistent with evidence according to which both oncometabolic transformation (e.g. IDH mutation leading to down-regulation of JHDM activity [10, 42]) and aging (e.g. down-regulation of SIRT6 [5, 19, 47]) induce loss of HDM and HDAC activity thus facilitating reprogramming.

### Heterogeneity and robustness of the refractory and plastic scenarios

In order to study the robustness of the refractory and plastic scenarios with respect to variations of the model parameters, *k*_*j*_ (see Table 1), we first generate an ensemble of parameter sets *θ* = (*k*_*j*_, *j* = 1, …, 16) compatible with simulated data for the epigenetic regulation systems. Such ensemble is generated using Approximate Bayesian Computation [48] (for further details see Section III in S1 File). Our approach is as follows. For each mode of epigenetic regulation, we have generated simulated data (denoted as “raw data” in Fig 2) using the stochastic simulation algorithm on the model defined by the transition rates Table 1. This simulated data will play the role of the experimental data, *x*_0_, to which we wish to fit our model. We consider two different data sets $x_{0_{d}}$ and $x_{0_{p}}$, corresponding to the differentiation gene (reaction rates from Table A in S1 File) and the pluripotency gene (reaction rates from Table B in S1 File), respectively. Each data set consists of 10 realisations and 25 time points per realisation. For each time point, *t*_*i*_, we consider two summary statistics: the mean over realisations, $\bar{x}\left(t_{i}\right)$, and the associated standard deviation, *σ*(*t*_*i*_). We then run the ABC rejection sampler method until we reach an ensemble of 10000 parameter sets which fit the simulated data, *x*_0_, within the prescribed tolerances for the mean and standard deviation. Fig 2(a) & 2(b) shows results comparing the reference (raw simulated) data to a sub-ensemble average (full posterior distributions are shown in Fig. C in S1 File, differentiation-promoting gene, and Fig. D in S1 File, pluripotency-promoting gene).

The above procedure provides us with an ensemble of parameter sets that are compatible with our raw data, i.e. such that they fit the data within the prescribed tolerances. The heterogeneity associated with the variability within this ensemble has a clear biological origin. The rates *k*_*j*_ are associated with the activity of the different enzymes that carry out the epigenetic-regulatory modifications (HDMs, HDACs, as well as, histone methylases (HMs) and histone acetylases (HACs)), so that variation in these parameters can be traced back to heterogeneity in the availability of cofactors, many of them of metabolic origin such as NAD+, which are necessary for these enzymes to perform their function (as illustrated in Fig 1).

**Fig 5:**
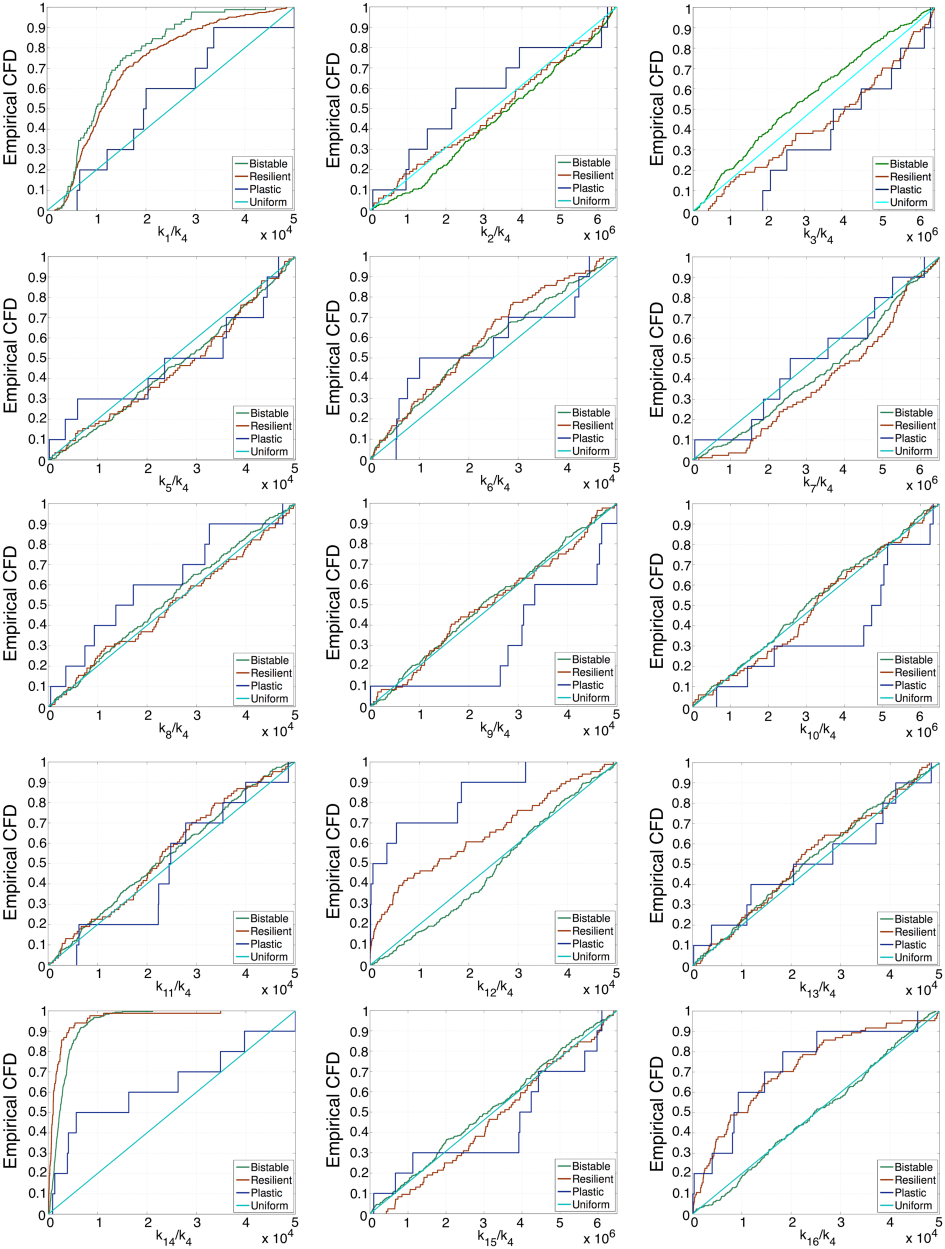
This figure shows the cumulative frequency distribution (CFD) for a sample consisting of the 401 differentiation gene ER parameter sets generated by ABC which best fit the synthetic data shown in Fig 2(a), i.e. SSA simulated data for the default stochastic ER differentiation system (see Table A in S1 File). Out of these 401 parameter sets, 94 satisfy the constraints associated with the differentiation epiphenotype. Amongst these, 10 are found to show plastic behaviour. The remaining 307 parameter sets generate bistability at *e*_*HDM*_ = *e*_*HDAC*_ = 1. Colour code: blue and red lines correpond to the CFD of the plastic and refractory differentiation epiphenotypes, respectively. Green lines correspond to the CFD of the parameters that generate bistability at *e*_*HDM*_ = *e*_*HDAC*_ = 1. Cyan lines correspond to the CFD of a uniform distribution, which we add for reference.

We first consider the differentiation ER system. In particular, we focus on the sub-ensemble of the 400 parameter sets that best fit the raw data. Within such sub-ensemble, we proceed to evaluate the robustness of the different scenarios we study. We consider that a particular scenario is sensitive to a specific parameter, *k*_*j*_, if its distribution is significantly different from the uniform distribution [49]. We first analyse the base-line scenario for the epigenetic regulation of a differentiation-regulated gene, namely, (i) when *e*_*HDM*_ = *e*_*HDAC*_ = 1, the regulatory system is mono-stable (only the open chromatin state is stable), and (ii) for *e*_*HDM*_ < 1, *e*_*HDAC*_ < 1 there exists a region of bistability. Out of all the parameter sets of the considered sub-ensemble, only 94 fulfill these requirements. We refer to these as the *viable set*. The remaining 307 are bistable at *e*_*HDM*_ = *e*_*HDAC*_ = 1, and they will be referred to as the *non-viable set*. In Fig 5, we present the cumulative frequency distributions (CFDs) of each *k*_*j*_ within both sets. The rationale for looking into this is that the requirements upon system behaviour associated with both sets should reflect themselves on the corresponding CFDs.

Regarding the viable set, we seek to assess which kinetic constants have distributions which deviate in a statistically significant manner from the uniform distribution [49]. Such parameters are deemed to be the essential ones for the ER system to exhibit the behaviour associated with the viable set. We perform this analysis by means of the Kolmogorov-Smirnov (KS) test [50, 51], which we use to compare our samples with the uniform distribution. According to such analysis, the kinetic constants *k*_1_, *k*_3_, *k*_6_, *k*_7_, *k*_12_, *k*_14_, and *k*_16_ are not uniformly distributed (p-values are reported in Table E in S1 File).

Nested within the viable set, there are parameter sets which exhibit plastic behaviour, as characterised by a phase diagram as per Fig 3(f). We thus continue by studying the plastic subset regarding both its frequency within the viable subset and further restrictions imposed on parameter variability. We first check the number of the plastic parameter sets within the viable set relative to the pluripotency-gene ER system defined by Table D in S1 File. Somehow unexpectedly, the plastic scenario is rare, but not exceptional: amongst the 94 parameter sets that we have identified as viable, 10 exhibit plasticity (see Fig 5 for their CFDs).

Further restrictions on parametric heterogeneity imposed by the plastic scenario are analysed regarding the variation of the CFDs of kinetic constants when compared to those associated with the whole viable subset. The results of KS analysis performed on the data shown in Fig 5 show that only the distributions of *k*_1_ (associated with recruited demethylation), *k*_9_ (unrecruited deacetylation), and *k*_14_ (recruited deacetylation) are significantly modified by the plasticity requirement (p-values reported in Table G in S1 File).

From a more mechanistic perspective, we observe that, within the plastic set, the mass of the CFDs of *k*_1_, *k*_9_ and *k*_14_ is displaced towards the large-value end of their intervals with respect to their behaviour within the full viable set. In other words, *k*_1_, *k*_9_ and *k*_14_ tend to be larger for plastic ER systems than for non-plastic, viable ER systems. In essence, we observe that ER systems exhibiting plastic behaviour tend to have increased activity in the enzymes performing histone deacetylation. This is consistent with recent evidence that aging decreases histone acetylation and promotes reprograming [5, 19, 47].

**Fig 6:**
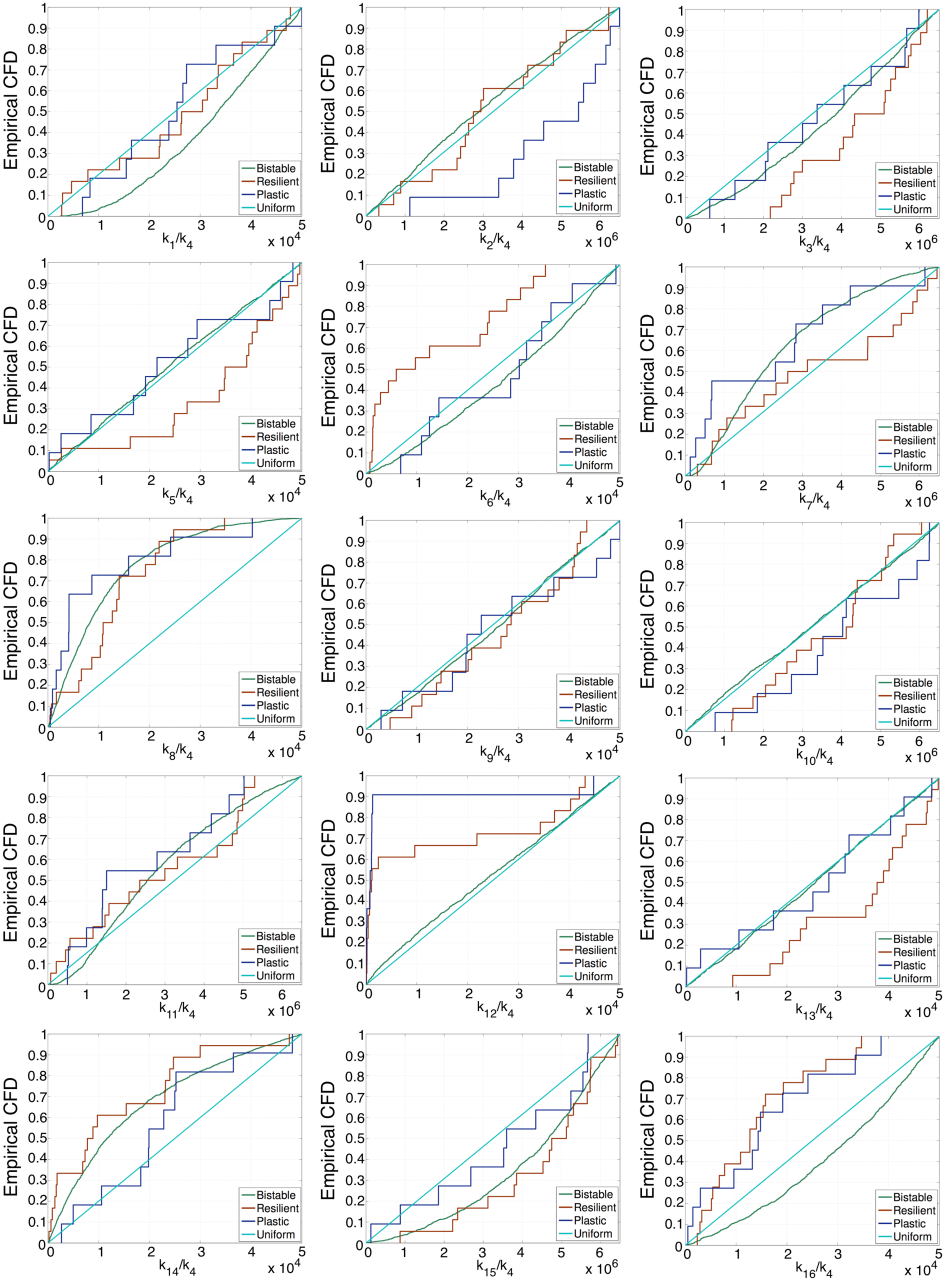
This figure shows the cumulative frequency distribution (CFD) for a sample consisting of the 1401 pluripotency gene ER parameter sets generated by ABC which best fit the synthetic data, i.e. SSA simulated data for the default stochastic ER pluripotency system (see Table B in S1 File). Out of these 1401 parameter sets, 29 satisfy the constraints associated with the pluripotency epiphenotype. Amongst these, 11 are found to show plastic behaviour. Another 1367 parameter sets generate bistability at *e*_*HDM*_ = *e*_*HDAC*_ = 1. The remaining 5 parameter sets are bistable at *e*_*HDM*_ = *e*_*HDAC*_ = 1 but they are rejected since their steady states do not correspond to open/closed situations. Colour code: blue and red lines correpond to the CFD of the plastic and refractory pluripotency epiphenotypes, respectively. Green lines correspond to the CFD of the parameters that generate bistability at *e*_*HDM*_ = *e*_*HDAC*_ = 1. Cyan lines correspond to the CFD of a uniform distribution, which we add for reference.

The same analysis has been conducted regarding the ensemble of parameter values generated using ABC for the pluripotency gene ER system (full posterior distribution in Fig. D in S1 File). The results of this analysis are shown in Fig 6. Detailed analysis using the KS test of the ensemble viable pluripotency ER systems shows that *k*_3_, *k*_8_, *k*_12_, *k*_14_, *k*_15_, and *k*_16_ are significantly constrained by the requirements of such scenario (i.e. their CDF departs significantly from the uniform distribution, as shown by the p-values from Table F in S1 File). We then move on to investigate further restrictions within the plastic set. We observe that only the CDFs associated with *k*_2_ and *k*_6_ are significantly different (p-values reported in Table H in S1 File). In both cases, values of *k*_2_ and *k*_6_ associated with plasticity are larger than in the general viable population. Both parameters are associated with demethylation activity.

Our ensemble analysis thus provides a rationale for the coupling between variations in the size of the pool of epigenetic cofactors and increased reprogramming in a heterogeneous cell population. A notable case in point is provided by metabolic changes during aging: those cells where key metabolites such as acetyl-CoA and NAD+ are less abundant lose acetylation capability (in our model, this is reflected through the dependence of histone-modifying enzyme activity on the concentration of these cofactors), leading to cells poised for reprogramming.

**Fig 7:**
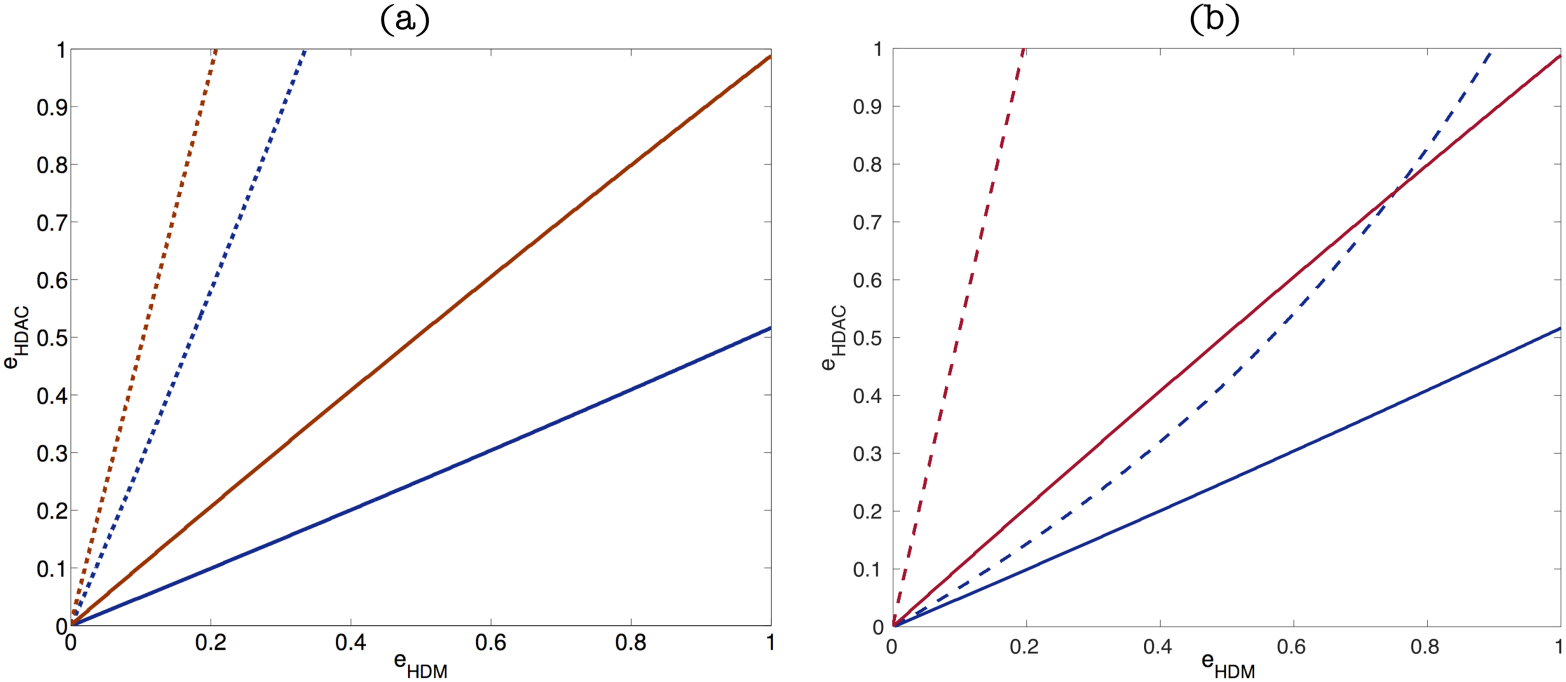
(a)This plot shows results regarding restoration of base-line behaviour by removal of plasticity by restoring acetylation activity. It shows the phase space corresponding to the ER system composed of a differentiation-promoting gene with parameter set given by *θ*′ with $k_{9}^{\prime }=k_{9}/4$ (see text for details) and a pluripotency-gene with parameters given by Table D in S1 File. This result demonstrates that by reducing deacetylation activity, we can drive the system off plastic behaviour and restore the normal situation as described by the base-line scenario. (b) This plot shows results regarding the appearance of the plastic behaviour by increasing deacetylation activity. Parameter values for the differentiation-promoting gene are given by *θ*′ with $k_{9}^{\prime }=3k_{9}$ and $k_{14}^{\prime }=3k_{14}$ (see text for details) and for the pluripotency-promoting gene are given by Table D in S1 File. This result shows an strategy to drive the system to the plastic scenario and hence, indicates how to obtain favourable scenarios for reprogramming.

This analysis provides a rationale for a strategy to interfere with the epigenetic regulatory system, regarding the ability to either drive the system away from plastic behaviour or to drive it to the plasticity scenario, while keeping it functional (i.e. within the restrictions of the base-line scenario). An example illustrating the effectiveness of this strategy is shown in Fig 7. Consider the viable set of the ER differentiation-promoting gene, Fig 5, which is neutral with respect to the value of *k*_9_: *k*_9_ remains uniformly distributed within the viable subset. By contrast, when plasticity is required, the admissible values of *k*_9_ accumulate mostly towards the large-value end. This suggests that decreasing the value of *k*_9_ might be a viable strategy to restore resilience. To check this, we consider the parameter set, *θ* = *k*_*j*_/*k*_4_, *j* = 1, …, 16, that gives rise to the plastic behaviour depicted in Fig 3(f) (Table C in S1 File, for the differentiation-promoting gene). We then analyse the effect of modifying the value of *k*_9_ for the differentiation-promoting gene on system behaviour. The new parameter set, $\theta^{\prime }=k_{j}^{\prime }/k_{4},\;j=1,\ldots ,16$, is such that $k_{9}^{\prime }=k_{9}/4$ and $k_{j}^{\prime }=k_{j}$ for all *j* ≠ 9 (*k*_*j*_ values as per Table C in S1 File). Parameter values for the pluripotency gene remain unchanged (as per Table D in S1 File). The corresponding phase space is shown in Fig 7(a). We observe that by reducing deacetylase activity in this fashion, the ER system reverts to resilient behaviour. This suggests that, by regulating the abundance of cofactors associated with (de)acetylation, we can drive the system off the plastic regime into the base-line behaviour.

Similarly, we can seek for complex, combined strategies to increase the robustness of plastic behaviour. An example of such strategy is shown in Fig 7(b). Based on the results of the KS test for the differentiation-promoting gene, we observe that deacetylation-related rates *k*_9_ and *k*_14_ are significantly increased in plastic scenarios. Taking parameter sets from a resilient scenario (Tables A & D in S1 File, which lead to a combined phase diagram qualititatively similar to that shown in Fig 3(d)) and modifying *k*_9_ and *k*_14_ for the differentiation-promoting gene so that $k_{9}^{\prime }=3k_{9}$ and $k_{14}^{\prime }=3k_{14}$ while keeping all the others at the same value, the resulting ER system corresponds to a plastic system. Futhermore, this combined strategy results in more robust plasticity (as compared to e.g. the case shown in Fig 3(f)), as measured by the area of the phase space region where reprogramming is feasible. This indicates that by combining the strategies suggested by the statistical analysis of the plastic sub-ensemble, we can find conditions for optimal conditions to achieve robust reprogramming. This, in turn, highlights the importance of cofactor levels, since as it has been shown in Fig 7, depending on its availability, the same ER system can be driven to the plastic or resilient state.

These strategies require close attention to be payed to the correlations between parameters. Parameters in complex systems biology models exhibit strong correlations which confer the system with essential properties such as *sloppiness*, which refers to the property exhibited by many multi-parameter systems biology models, whereby the system’s behaviour is insensitive to changes in parameter values except along a small number of parameter combinations [35]. In order to quantify such correlations, we have used hierarchical clustering. The results are shown in Fig. E(a) & E(b) in S1 File for the base-line and the plastic scenarios of the differentiation-regulating ER system, respectively. Not unexpectedly, we observe that, with respect to the base-line scenario, correlations substantially change when the plastic scenario is considered. Although the strategies illustrated in the results shown in Fig 7 changed one or two parameters alone independently of all the others, more general situations will require to closely monitor these correlations to understand which combinations of parameters are relevant to control the system’s behaviour [35].

## Discussion

**Fig 8:**
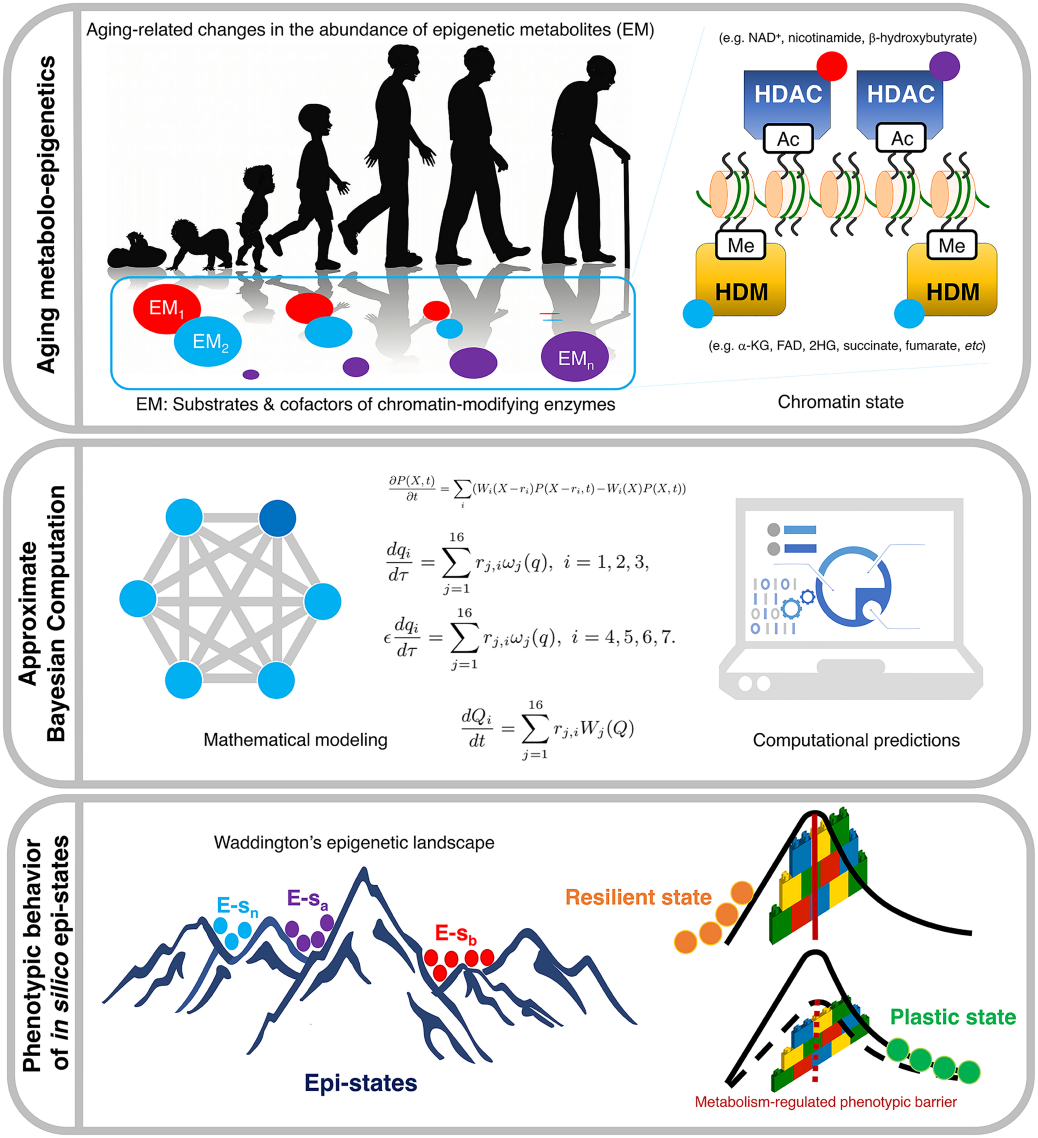
Epigenetic regulation of cell fate reprogramming in aging and disease: A predictive computational model. Cell reprogramming, a process that allows differentiated cells to re-acquire stem-like properties, is increasingly considered a critical phenomenon in tissue regeneration, aging, and cancer. In light of the importance of metabolism in controlling cell fate, we designated a computational model capable of predicting the likelihood of cell reprogramming in response to changes in aging-related epigenetic metabolites (EM). Our first-in-class Approximate Bayesian Computation (ABC) approach integrates the biochemical basis of aging-driven metabolite interaction with chromatin-modifying enzymes to predict how aging-driven metabolic reprogramming could alter cell state transitions via reorganisation of chromatin marks without affecting the shape of the Waddingtonian epigenomic landscape. Our predictive mathematical model improves our understanding of how pathological processes that involve changes in cell plasticity, such as tissue repair and cancer, might be accelerated or attenuated by means of metabolic reprogramming-driven changes on the height of phenotypic transitioning barriers.

We here provide computational evidence for the role of stochastic translation of epigenetic cofactors into resilient/plastic cell states via ER systems as a mechanistic facilitator of cellular aging, and its reversal. When changes in levels of such cofactors operate as regulators of the kinetic parameters associated with chromatin-modifying enzymes such as HDMs and HDACs, the ensemble of ER configurations reveals the occurrence of cell-to-cell phenotypic variability in terms of different epi-states (see Fig 8). This model provides a rationale for the responsiveness of cellular phenotypes to metabolic signals, as metabolic pools serve as epigenetic cofactors. The metabolic control of epigenetic landscapes and cell state transitions might therefore operate as a common hub capable of facilitating the pathogenesis of aging-related diseases including cancer.

Several layers of molecular communication exist between cell metabolism and chromatin remodelling [16, 52–56]. A first layer of metabolo-epigenetic regulation includes metabolites/nutrient-responsive TF-dependent transcriptional regulation of chromatin regulators (HMT, HAT, DNMTS, etc), which can lead to global changes on chromatin structure. Second, metabolites can modulate chromatin modifications at specific genomic loci by affecting the activity/localisation of proteins that recruit or regulate chromatin-modifying enzymes during, for example, transcriptional activation phenomena. Third, chromatin-modifying enzymes employ many metabolites as donor substrates and cofactors, and changes in levels of these *bona fide* epigenetic metabolites can in turn lead to changes not only in the global status of chromatin modifications but also to gene specific regulation under different metabolic conditions.

Our mathematical model only incorporates the third such layer through cofactor-induced heterogeneity. Because any metabolic input has the potential to affect various chromatin marks via its effects on transcription, our model ignored metabolic regulation of TF activity. In contrast to other metabolically-regulated enzymatic activities such as phosphorylation in which the substrate (ATP) is present in cellular concentrations far greater than the enzyme *K*_*m*_ values, i.e., the concentration of metabolite at half maximum velocity of enzyme-mediated reaction, the physiological cellular concentrations of donors and cofactors that are employed by histone-modifying enzymes (e.g., organic ketoacids such as the demethylase cofactor *α*-ketoglutarate for HDMs or the NAD+ deacetylase cofactor for HDACs) are close to HDM and HDAC Km values [16, 57]; consequently, based solely on the intrinsic biochemical characteristics of chromatin-modifying enzymes such as HDMs and HDACs, small fluctuations in the concentrations of such metabolites could significantly alter HDM and HDAC activities, either increasing or decreasing their respective histone-modifying activities. This layer of metabolo-epigenetic regulation is commonly viewed as a direct link from cell metabolism to chromatin-modification status, which could be mathematically modelled and tested as has been confirmed in our current computational model (see Fig 8).

Evidence accumulates demonstrating that differing metabolomes can be found in distinct cell states, thereby suggesting how changes in metabolism can impact and probably specify cell fate via alteration of the chromatin landscape [58–63]. Yet, there is a scarcity of examples showing that metabolic changes can restructure the epigenetic landscape and lead to different cell states regardless of other global changes in cell physiology occurring in response to this variation in metabolite levels. Our findings support the notion that changes in the abundances of certain metabolites would alter specific chromatin marks, thereby determining both the stability of cell types and the probability of transitioning from one epi-state to another [64]. Our model infers that such a change in metabolite level would be sufficient to either impede or allow cell epi-state transitions by regulating the height of the phenotypic barriers in the context of Waddington’s landscape (Fig 8). However, we should acknowledge that the necessary involvement of cellular metabolism on the structure of the epigenetic landscape will require the experimental coupling of defined metabolic conditions with epigenome editing systems (e.g., CRISPR-Cas9) capable of targeting specific histone PTMs playing important roles in chromatin structure [65].

Our ensemble approach provides mechanistic support to the notion that emergence of the cellular and molecular hallmarks of aging including cancer might result from a metabolically driven loss of epigenetic resilience. Flavahan et al. [57] have recently proposed that non-genetic stimuli including aging and metabolic insults can induce either overly restrictive chromatin states, which can block tumor-suppression and/or differentiation programs, or overly permissive/plastic chromatin states, which might allow normal and cancer cells to stochastically activate oncogenic programs and/or nonphysiologic cell fate transitions. Our ensemble approach provides a framework that supports heterogeneity of epigenetic states as an engine that facilitates cancer hallmarks and other aging diseases. On the one hand, the ability of resilient states to maintain large epigenetic barriers refractory to non-physiologic cell fate transitions might explain why the NAD+-dependent HDAC/sirtuin pathway is one of the few mechanisms described to mediate the correction or resetting of the abnormal chromatin state of aging cells induced by calorie restriction, the most robust life span-extending and cancer preventing regimen [2, 66–68]. On the other hand, the ability of plastic states to lower epigenetic barriers, and increase the sensitivity of primed cells to undergo reprogramming-like events leading to loss of cell identity is consistent with the ability of certain metabolites to promote oncogenesis by epigenetically blocking the HDM-regulated acquisition of differentiation markers [17, 69–71].

The traditional view of cancer formation (i.e., the Knudson model [72]) exclusively involves the binary acquisition and accumulation of genetic alterations as the principal driver mechanism for the age-dependency of multistage cancer development. Our ensemble approach suggests an alternative, namely, that oncogenic chromatin aberrations might also occur via purely epigenetic stimuli. Our model shows that, nested within the ensemble of ER systems, those that prime cells for reprogramming exhibit properties associated with age-induced epigenetic dis-regulation [73, 74]. Aging-responsive ER reprogramming might thus operate in a more progressive and graded manner to increase cancer susceptibility without the need to induce genetic mutations. Our ensemble model is mechanistically consistent with the fact that those cancers in which the sole presence of epigenetic metabolites (e.g., oncometabolites) suffices to stabilise undifferentiated cellular states by preventing demethylation of genes implicated in differentiation have accelerated models of oncogenesis [44, 75–82]. Whereas the epigenetic signature of adult somatic cells must be partially and acutely erased to adopt a more plastic epigenome, such cellular plasticity, which might occur via metabolically driven epigenetic activation of promoter regions of pluripotency genes, could impose a chronic, locked gain of stem cell-like states disabled for reparative differentiation.

The existence of metabolism-permissive resilient and plastic epigenetic landscapes might have predictive power on the susceptibility of a cell to lose its normal cellular identity through reprogramming-like resetting phenomena. The beneficial or deleterious decision paths during the maintenance of cell and tissue homeostasis might be closely related to the ability of epigenetic landscapes to modulate the intrinsic responsiveness to reprogramming cellular identity. The incapability of finishing cellular reprogramming, or at least to increase cellular epigenetic plasticity, might impede tissue self-repair in response to injury, stress, and disease, thus driving the observed aging phenotypes. Accordingly, the infliction of chronic injury and the aging phenotype have been shown to render tissues highly permissive to in vivo reprogramming [47] while the cyclic, transient expression of reprogramming factors has recently been shown to increase lifespan in a murine model of premature aging via remodeling of the chromatin landscape [7]. Because our model suggests that the fine-tuning of metabolic epigenetic cofactors might direct plastic epigenetic states to re-enter into epigenetic resilience, and vice versa, it would be relevant to experimentally evaluate whether specific metabolic interventions might either mimic transient reprogramming and revert some age-associated features without promoting complete undifferentiation, or prevent the occurrence of unrestricted/uncontrolled plasticity in chronically injured tissues such as those occurring in aging and cancer.

In summary, by integrating the ability of chromatin epigenetic modifiers to function as sensors of cellular metabolism, our ensemble model provides computational support to the notion that a metabolism-responsive loss of epigenetic resilience might mechanistically facilitate cellular aging. The stochastic translation of metabolic signals into resilient/plastic cell states via ER systems might be viewed as a metabolo-epigenetic dimension that not only facilitates cellular aging, but that also offers new therapeutic and behavioural avenues for its reversal. Our findings strongly suggest that the development of predictive mathematical models and computational simulation platforms capable of operatively integrate the metabolic control of epigenetic resilience and plasticity and its combination with confirmatory lab-based testing might accelerate the discovery of new strategies for metabolically correcting the aberrant chromatin structure that affects cellular identity and epi-state transitions in aging and aging-related diseases.

## Supporting information

S1 File Online supplemental information. (PDF)
