# Supplementary material for: Epigenetic regulation of cell fate reprogramming in aging and disease: A predictive computational model
Source: PLoS Comput Biol. 2018 Mar 15;14(3):e1006052. doi: 10.1371/journal.pcbi.1006052 (PMC5871006; doi:10.1371/journal.pcbi.1006052)
Supplement: S1 File — (PDF) [file pcbi.1006052.s001.pdf]

Epigenetic regulation of cell fate reprogramming in aging and disease: A predictive computational model
Supporting information

Núria Folguera-Blasco,[1,2] Elisabet Cuyàs,[3,4] Javier A. Menéndez,[3,4,5] and Tomás Alarcón[6,1,2,7]

[1]Centre de Recerca Matemàtica, Edifici C, Campus de Bellaterra, 08193 Bellaterra (Barcelona), Spain.
[2]Departament de Matemàtiques, Universitat Autònoma de Barcelona, 08193 Bellaterra (Barcelona), Spain.
[3]Molecular Oncology Group, Girona Biomedical Research Institute (IDIBGI), Girona, Spain.
[4]MetaboStem, Barcelona, Spain.
[5]ProCURE (Program Against Cancer Therapeutic Resistance), Metabolism and Cancer Group, Catalan Institute of Oncology, Girona, Spain.
[6]ICREA, Pg. Lluís Companys 23, 08010 Barcelona, Spain.
[7]Barcelona Graduate School of Mathematics (BGSMath), Barcelona, Spain.

(Dated: March 3, 2018)

In this appendix, we present supplementary materials regarding the methods of our manuscript *Epigenetic regulation of cell fate reprogramming in aging and disease: A predictive computational model*. The materials presented below further contains a summary of the Approximate Bayesian Computation method, as well as further simulation results, statistical analysis, and materials (such as parameter values). We also include Supplementary Figures which complement the results discussed in the main text.

I. FURTHER SIMULATION RESULTS REGARDING THE BIFURCATION ANALYSIS

We present simulation results verifying the bifurcation analysis of the equations discussed in Section Variation in the abundance of HDM and HDAC drives epigenetic switch, of the main text. In particular, we explicitly show the existence of the hysteresis cycles predicted by our bifurcation analysis. For concreteness, since the behaviour of both differentiation- and pluripotency-promoting genes is qualitatively similar, we specifically focus on simulations of the differentiation-promoting gene. Differences with the pluripotency case only concern the quantitave value of the critical (bifurcation) points, not the behaviour of the system.

Fig. A shows simulation results where we have set the number of HDAC enzymes, v_0, to $v_0 = E$ (see Table A). Note that according to the description in the main text, this is equivalent to fixing the HDAC concentration to $e_{HDAC} = 1$, since $e_{HDAC} = \frac{v_0}{E}$. We then vary z_0 which, according to the main text, where we define $e_{HDM} = \frac{z_0}{E}$, is the same as varying HDM concentration. Fig. A shows results regarding the empirical distribution of x_3, $P(x_3)$, where $x_3 \equiv X_3(t_{inf})/S$ with t_{inf} the duration of the simulation which is taken long enough so that the system settles onto its quasi-steady state starting from prescribed initial conditions $X_i(t = 0)$. In order to ascertain whether the system exhibits the hysteresis cycle predicted by our bifurcation analysis as z_0 changes (see Fig. 3(c), main text), we first set an initial condition with $X_1(t = 0) = 0$, $X_2(t = 0) = 0.9S$, $X_3(t = 0) = 0.1S$. This allows us to explore the behaviour of the system along the lower stable branch (corresponding to closed chromatin) of the diagram Fig. 3(c) of the main text. Similarly, by setting initials conditions to $X_1(t = 0) = 0$, $X_2(t = 0) = 0.01S$, $X_3(t = 0) = 0.99S$, in Fig. A(b) we trace the behaviour of the system along the upper stable branch (associated with open chromatin).

Fig. A(a) shows that, for the prescribed initial condition, the system exhibits a unimodal distribution around the closed chromatin state for small values of z_0. As z_0 increases and approaches the critical value where the closed chromatin state ceases to exist, fluctuations increase (as shown by the bimodal behaviour of $P(x_3)$) thus heralding the onset of a phase transition. Beyond this point, $P(x_3)$ exhibits unimodal behaviour around the open chromatin state. These results, including the value of the critical point (which in the simulations is formally characterised by a divergence of the variance of x_3), are in agreement with those obtained from the bifurcation analysis (see Fig. 3(c), main text).

In Fig. A(b) we trace the other half of the hysteresis cycle. By setting initial conditions to $X_1(t = 0) = 0$, $X_2(t = 0) = 0.01S$, $X_3(t = 0) = 0.99S$, $P(x_3)$ exhibits unimodal behaviour around the open chromatin state for larger values of z_0. As z_0 is reduced and approaches the critical value for which the open chromatin state ceases to exist, fluctuations are again observed to increase, i.e. $P(x_3)$ becomes bimodal around the critical point. Beyond this point, $P(x_3)$ recovers unimodal behaviour but peaked around the closed chromatin state. Results, including the value of the critical point, are again in excellent agreement with the bifurcation analysis (see Fig. 3(c), main text).

Fig. B shows the same results regarding the verification of the bifurcation analysis shown in Fig. 3(e), main text. Here, we have set the number of HDM enzymes, z_0, to $z_0 = 0.2E$ (see Table A). We then simulate the behaviour of the system as the number of HDAC molecules, v_0, is changed. We observe the same excellent agreement between simulations and bifurcation analysis as we do between the numerical and analytical results shown in Figs. A and 3(c) (main text), respectively.

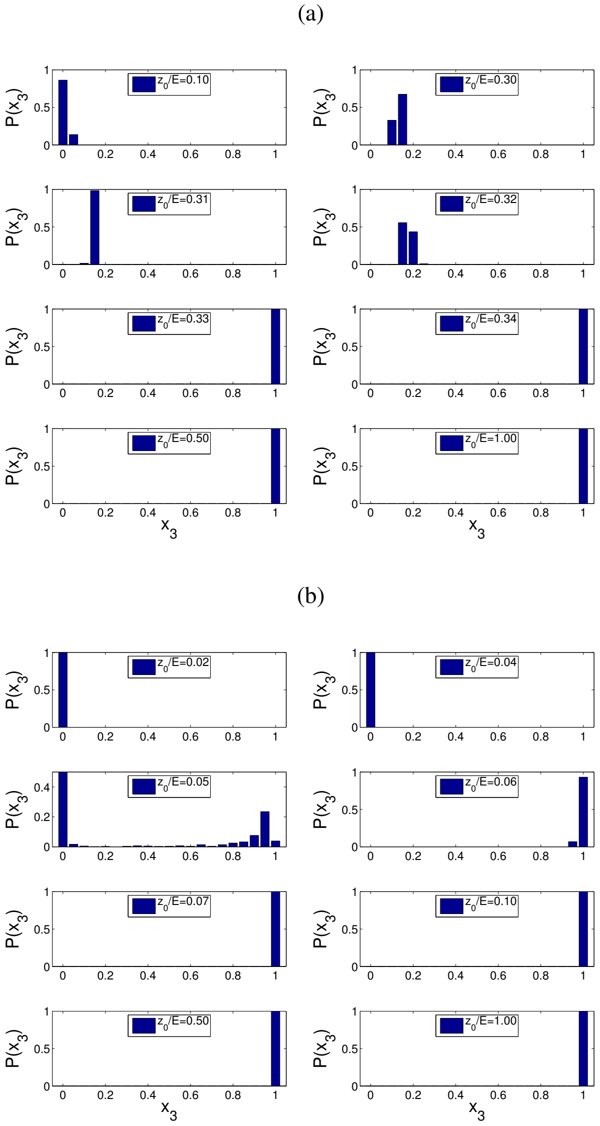

FIG. A: Simulation results corresponding to Fig. 3(c), main text. Each histogram shown in this figure is the result of 1000 realisations of the stochastic process prescribed by the rates shown in Table 1, main text. Parameter values are obtained from Tables A and 2, main text. See main text for more details.

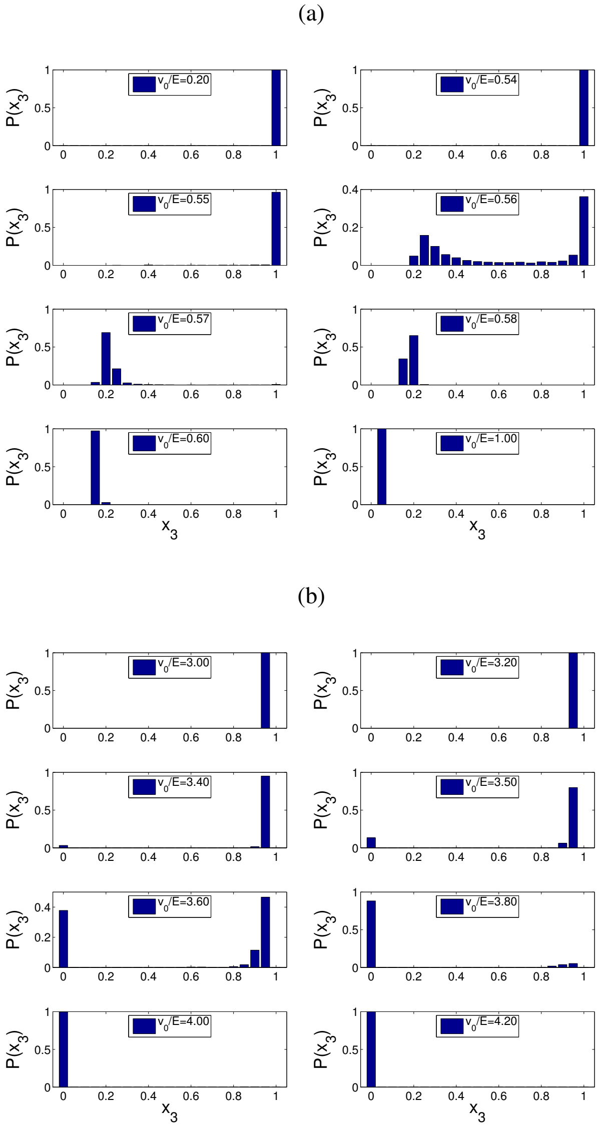

FIG. B: Simulation results associated with Fig. 3(e), main text. Each histogram shown in this figure is the result of 1000 realisations of the stochastic process prescribed by the rates shown in Table 1, main text. Parameter values are obtained from Tables A and 2, main text. See main text for more details.

II. REFERENCE PARAMETER VALUES: REFRACTORY & PLASTIC SCENARIOS

Here we give the sets of parameter values used to produce the phase diagrams Fig. 3 of the main text, associated both with the reprogramming-resilient phenotype (Fig. 3(d) of the main text) and the phenotype with elevated plastic potential (Fig. 3(f) of the main text). These sets of parameter values have been chosen with the same viability criteria as those given in Section Parameter values & ensemble generation of the main text, namely, that the mean-field limit has a single stable steady state corresponding to the open (closed) epigenetic state for the differentiation (pluripotency) gene. Besides this, we further require that, for the reprogramming-resilient phenotype there is no overlap of the bistability regions, whereas for the plastic phenotype we require the area between the solid red line and the dashed blue line (see Fig. 3(f) of the main text) to be positive. The reference parameter values are given in Tables A, B, C and D.

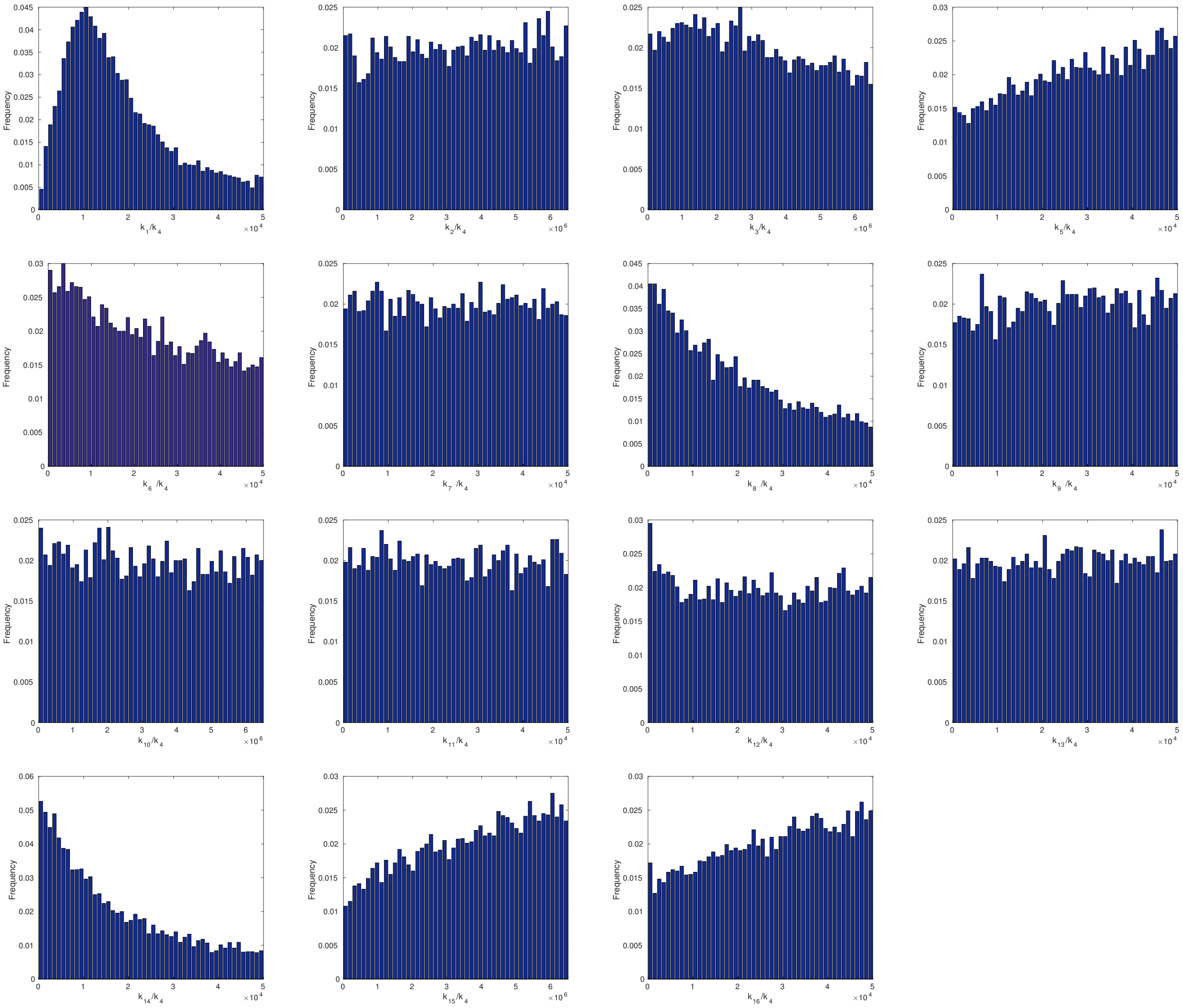

FIG. C: Differentiation gene. $\epsilon_1 = 0.6$ and $\epsilon_2 = 1$. $z_0 = v_0 = E = 5$ and $S = 250$.

III. SENSITIVITY ANALYSIS AND ROBUSTNESS OF THE PLASTIC SCENARIO

As a first step towards the analysis of the robustness of the plastic scenario associated with the situation depicted in the bifurcation diagram shown in Fig. 3(f) of the main text, we have carried out an exhaustive parameter sensitivity analysis. In particular, we are interested in which of the reaction rates k_j are critical to obtain the epigenetic regulation associated with the differentiation gene (i.e. the steady state behaviour corresponding to open chromatin) and which ones are critical for the epigenetic regulation corresponding to the pluripotency gene (i.e. the steady state behaviour corresponding to closed chromatin). We have used an Approximate Bayesian Computation (ABC) method [1] to perform our exploration of parameter space.

ABC methods have been devised to tackle those inference problems for which the estimation of the likelihood function is computationally too demanding. Let $\theta = (k_1, \ldots, k_{16})$ be the vector whose components are the parameters to be estimated and x be the data. The general aim is to approximate the so-called posterior distribution, $\pi(\theta|x)$, i.e. the conditional probability of θ given the data, from a prior distribution of the parameters, $\pi(\theta)$. In general, the posterior is given by $\pi(\theta|x) \sim f(x|\theta)\pi(\theta)$, where $f(x|\theta)$ is the likelihood function. All ABC methods follow the same generic procedure:

- Sample a candidate sequence of parameters, θ, from the proposed prior distribution, $\pi(\theta)$.
- Sample or simulate a data set x from the model represented by the conditional probability density $f(x|\theta)$.
- Compare the simulated data set, x, to the experimental data, x_0, according to some distance function, $d(x, x_0)$.

Rescaled parameter	Scaling parameter	Units	Reference
$\kappa_1 = 200$		dimensionless	–
$\kappa_2 = 100$		dimensionless	–
$\kappa_3 = 50$		dimensionless	–
$\kappa_5 = 1$		dimensionless	–
$\kappa_6 = 200$		dimensionless	–
$\kappa_7 = 10$		dimensionless	–
$\kappa_8 = 100$		dimensionless	–
$\kappa_9 = 200$		dimensionless	–
$\kappa_{10} = 100$		dimensionless	–
$\kappa_{11} = 0.1$		dimensionless	–
$\kappa_{12} = 1$		dimensionless	–
$\kappa_{13} = 1$		dimensionless	–
$\kappa_{14} = 200$		dimensionless	–
$\kappa_{15} = 100$		dimensionless	–
$\kappa_{16} = 100$		dimensionless	–
	$E = 100$		–
	$S = 5000$		–

TABLE A: Reference parameter values used for the epigenetic regulation of a differentiation-promoting gene, reprogramming-resilient phenotype (Fig. 3(d) of the main text).

Rescaled parameter	Scaling parameter	Units	Reference
$\kappa_1 = 200$		dimensionless	–
$\kappa_2 = 100$		dimensionless	–
$\kappa_3 = 10$		dimensionless	–
$\kappa_5 = 1$		dimensionless	–
$\kappa_6 = 10$		dimensionless	–
$\kappa_7 = 100$		dimensionless	–
$\kappa_8 = 100$		dimensionless	–
$\kappa_9 = 200$		dimensionless	–
$\kappa_{10} = 100$		dimensionless	–
$\kappa_{11} = 10$		dimensionless	–
$\kappa_{12} = 1$		dimensionless	–
$\kappa_{13} = 1$		dimensionless	–
$\kappa_{14} = 100$		dimensionless	–
$\kappa_{15} = 100$		dimensionless	–
$\kappa_{16} = 100$		dimensionless	–
	$E = 100$		–
	$S = 5000$		–

TABLE B: Reference parameter values used for the epigenetic regulation of a pluripotency-promoting gene, reprogramming-resilient phenotype (Fig. 3(d) of the main text).

Rescaled parameter	Scaling parameter	Units	Reference
$\kappa_1 = 200$		dimensionless	–
$\kappa_2 = 100$		dimensionless	–
$\kappa_3 = 30$		dimensionless	–
$\kappa_5 = 1$		dimensionless	–
$\kappa_6 = 100$		dimensionless	–
$\kappa_7 = 50$		dimensionless	–
$\kappa_8 = 100$		dimensionless	–
$\kappa_9 = 200$		dimensionless	–
$\kappa_{10} = 100$		dimensionless	–
$\kappa_{11} = 0.1$		dimensionless	–
$\kappa_{12} = 1$		dimensionless	–
$\kappa_{13} = 1$		dimensionless	–
$\kappa_{14} = 200$		dimensionless	–
$\kappa_{15} = 80$		dimensionless	–
$\kappa_{16} = 70$		dimensionless	–
	$E = 100$		–
	$S = 5000$		–

TABLE C: Reference parameter values used for the epigenetic regulation of a differentiation-promoting gene, plastic phenotype (Fig. 3(f) of the main text).

Rescaled parameter	Scaling parameter	Units	Reference
$\kappa_1 = 200$		dimensionless	–
$\kappa_2 = 100$		dimensionless	–
$\kappa_3 = 10$		dimensionless	–
$\kappa_5 = 1$		dimensionless	–
$\kappa_6 = 50$		dimensionless	–
$\kappa_7 = 100$		dimensionless	–
$\kappa_8 = 100$		dimensionless	–
$\kappa_9 = 200$		dimensionless	–
$\kappa_{10} = 100$		dimensionless	–
$\kappa_{11} = 8$		dimensionless	–
$\kappa_{12} = 1$		dimensionless	–
$\kappa_{13} = 1$		dimensionless	–
$\kappa_{14} = 100$		dimensionless	–
$\kappa_{15} = 100$		dimensionless	–
$\kappa_{16} = 100$		dimensionless	–
	$E = 100$		–
	$S = 5000$		–

TABLE D: Reference parameter values used for the epigenetic regulation of a pluripotency-promoting gene, plastic phenotype (Fig. 3(f) of the main text).

If $d(x, x_0) \leq \epsilon$, where ϵ is an a priori prescribed tolerance, then θ is accepted.

The result of this algorithm is a sample of parameters from a distribution $\pi(\theta | d(x, x_0) \leq \epsilon)$.

In our case, we have used the following version of the ABC rejection sampler method [1]:

1. Sample θ^* from $\pi(\theta) = \prod_j \pi_j(k_j)$ where $\pi_j(k_j) = U(0, 6.5 \cdot 10^6)$ for k_j, $j = 2, 3, 7, 10$ and 15, and $\pi_j(k_j) = U(0, 5 \cdot 10^4)$, otherwise, for the differentiation-promoting gene, and $\pi_j(k_j) = U(0, 6.5 \cdot 10^6)$ for k_j, $i = 2, 3, 7, 10, 11$ and 15, and $\pi_j(k_j) = U(0, 5 \cdot 10^4)$, otherwise, for the pluripotency-promoting gene.

2. Simulate data set, x^*, from the Master Equation with transition rates as per Table 1, main text, using Gillespie's stochastic simulation algorithm. We generate 10 realisations and collect data at times t_i, $i = 1, \ldots, 25$, corresponding to the raw data time points.

3. For each time point, t_i, we compute two summary statistics: the mean over the 10 realisations, $\overline{x^*}(t_i)$, and the associated standard deviation, $\sigma^*(t_i)$.

4. If $\sum_{i=1}^{25} \left(\overline{x^*}(t_i) - \overline{x_0}(t_i)\right)^2 \leq \epsilon_1$ and $\sum_{i=1}^{25} \left(\sigma^*(t_i) - \sigma_0(t_i)\right)^2 \leq \epsilon_2$ hold, θ^* is accepted,

where x_0 denotes the experimental data.

5. Go back to Step 1.

We run this algorithm until the number of accepted parameter sets reaches 10000. This method has been used to generate an ensemble of differentiation epigenetic regulation systems (see Fig. C) and an ensemble of pluripotency epigenetic regulation systems (shown in Fig. D).

IV. KOLMOGOROV-SMIRNOV ANALYSIS

In order to analyse the statistical significance of our results of Section Heterogeneity and robustness of the refractory and plastic scenarios (main text) regarding the shapes of the distributions of the kinetic parameters, k_j, associated with the different scenarios we consider (refractory vs plastic), we resort to a well-known method, namely, the Kolmogorov-Smirnov (KS) test [2, 3]. The KS test is a non-parametric test which allows to evaluate the equality of continuous probability distributions. This test can be used both to compare an empirically obtained sample with a reference probability distribution, or to compare two empiricial samples. In other words, the KS test allows us to tell whether two distributions are the same within the level of confidence we desire. Since this is a well-known technique, we will not go into its details and we will report our results. Interested readers are referred to the specialised literature for details [2, 3]. Throughout this section, we impose a level of confidence of 95 %.

A. Comparing the viable subset with the uniform distribution

The first test we are interested in carrying out consists on checking which kinetic constants, k_j, exhibit a non-uniform distribution within the viable subset. These parameters are the ones deemed to play a substantial role in the associated behaviour (i.e. viable (base-line) conditions of the differentiation/pluripotency ER system) [4]. The null hypothesis for the test is therefore whether the empirical cumulative frequency distribution (CFD) of each k_j is equal to the uniform. Since we are using a confidence interval of 95%, whenever the p-value is larger than 0.05 the null hypothesis cannot be rejected, i.e. the parameter is deemed to be uniformly distributed. As we mention in Section Heterogeneity and robustness of the refractory and plastic scenarios (main text), the null hypothesis is rejected only for k_1, k_3, k_6, k_7, k_{12}, k_{14}, and k_{16} (differentiation-promoting gene) and for k_3, k_8, k_{12}, k_{14}, k_{15} and k_{16} (pluripotency-promoting gene). The reported p-values are given in Table E and Table F, respectively.

B. Comparing the plastic sets with the viable subset

We continue our analysis by testing the CFDs of the kinetic constants when we consider those parameter sets that exhibit plastic behaviour. We analyse which parameters have different distributions when compared to their distributions within the viable set. These parameters are the ones deemed essential for the associated behaviour (i.e. plastic behaviour). The null hypothesis for the test is therefore whether the empirical CFD of each k_j for the plastic sets is equal to their empirical distributions within the whole viable set. Since we are using a confidence interval of 95%, whenever the p-value is larger than 0.05 the null hypothesis cannot be rejected, i.e. the parameter is deemed to be uniformly distributed. As reported in Section Heterogeneity and robustness of the refractory and plastic scenarios (main text), the null hypothesis is rejected only for k_1, k_9 and k_{14} (differentiation-promoting gene) and for k_2 and k_6 (pluripotency-promoting gene). The reported p-values are given in Table G and Table H, respectively.

Parameter	p-value
k_1	$1.273016 \cdot 10^{-13}$
k_3	0.015373
k_6	0.002362
k_7	0.005169
k_{12}	$1.969483 \cdot 10^{-8}$
k_{14}	$2.467713 \cdot 10^{-51}$
k_{16}	$4.555466 \cdot 10^{-11}$

TABLE E: Reported p-values for the parameters for which the hypothesis that they are distributed uniformly was rejected (diff.-promoting gene). Confidence interval of 95%. The p-values for the rest of the kinetic constants are all larger than 0.05.

Parameter	p-value
k_3	0.048676
k_8	$1.025119 \cdot 10^{-5}$
k_{12}	$1.489366 \cdot 10^{-12}$
k_{14}	$8.060631 \cdot 10^{-4}$
k_{15}	0.026272
k_{16}	$3.781311 \cdot 10^{-4}$

TABLE F: Reported p-values for the parameters for which the hypothesis that they are distributed uniformly was rejected (plurip.-promoting gene). Confidence interval of 95%. The p-values for the rest of the kinetic constants are all larger than 0.05.

Parameter	p-value
k_1	0.023493
k_9	0.037880
k_{14}	$1.492134 \cdot 10^{-4}$

TABLE G: Reported p-values for the parameters for which the hypothesis that they have the same distribution as that within the viable subset was rejected, diff.-promoting gene. Confidence interval of 95%. The p-values for the rest of the kinetic constants are all larger than 0.05.

V. SUPPLEMENTARY FIGURES

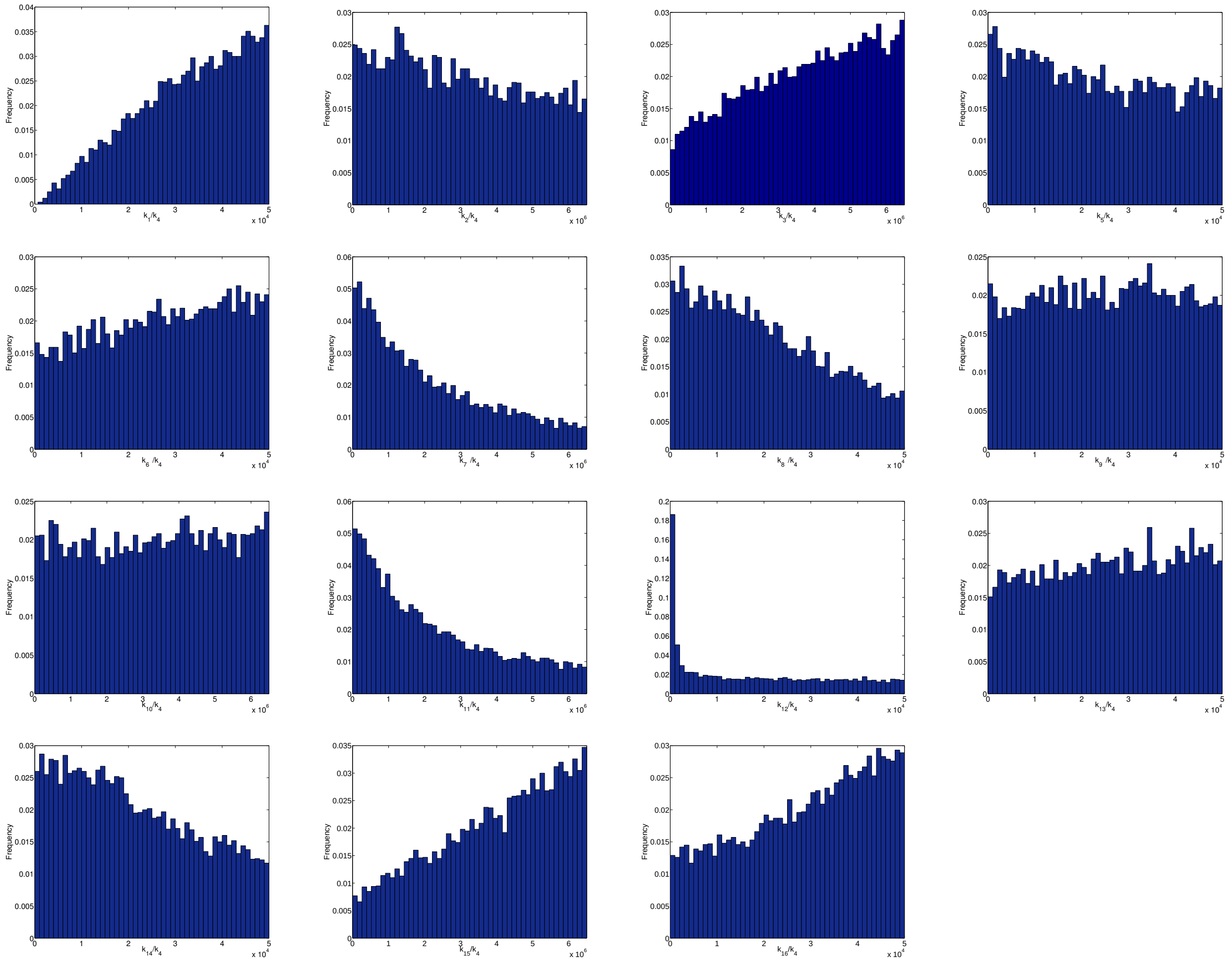

FIG. D: Pluripotency gene. $\epsilon_1 = 0.4$ and $\epsilon_2 = 1$. $z_0 = v_0 = E = 5$ and $S = 250$.

Parameter	p-value
k_2	0.0310
k_6	0.0425

TABLE H: Reported p-values for the parameters for which the hypothesis that they have the same distribution as that within the viable subset was rejected, plurip.-promoting gene. Confidence interval of 95 %. The p-values for the rest of the kinetic constants are all larger than 0.05.

[1] T. Toni, D. Welch, N. Strelkowa, A. Ipsen, and M. P. H. Stumpf, J. R. Soc. Interface. **6**, 187 (2009).

[2] D. A. Darling, Ann. Math. Statist. **28**, 823 (1957).

[3] A. Clauset, C. R. Shalizi, and M. E. J. Newman, SIAM Review **51**, 661 (2009).

[4] S. Filippi, C. P. Barnes, P. D. W. Kirk, T. Kudo, K. Kunida, S. S. McMahon, T. Tsuchiya, T. Wada, S. Kuroda, and M. P. H. Stumpf, Cell Reports **15**, 2524 (2016).

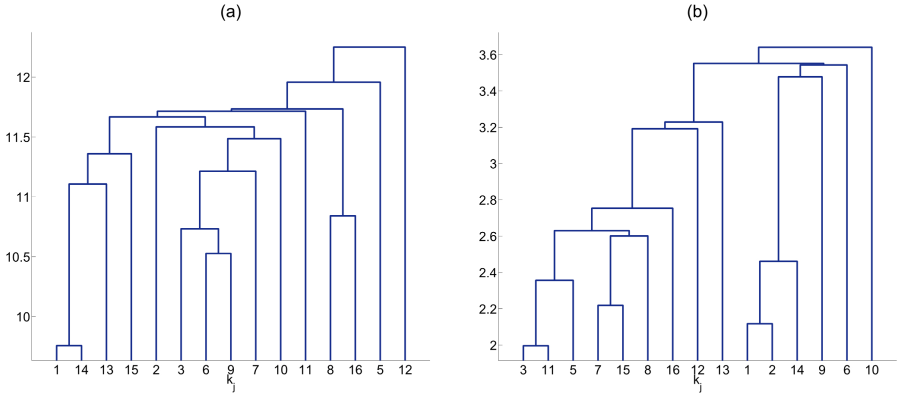

FIG. E: Plots showing hierarchical clustering analysis for the parameter sets that satisfy the base-line scenario (plot (a)) and the plastic scenario (plot (b)) of the differentiation-regulating ER system, respectively.
